# Mitochondrial Sirt3 contributes to the bone loss caused by aging or estrogen deficiency

**DOI:** 10.1172/jci.insight.146728

**Published:** 2021-05-24

**Authors:** Wen Ling, Kimberly Krager, Kimberly K. Richardson, Aaron D. Warren, Filipa Ponte, Nukhet Aykin-Burns, Stavros C. Manolagas, Maria Almeida, Ha-Neui Kim

**Affiliations:** 1 Center for Musculoskeletal Disease Research and Center for Osteoporosis and Metabolic Bone Diseases, Division of Endocrinology, Department of Internal Medicine,; 2 Division of Radiation Health, Department of Pharmaceutical Sciences, University of Arkansas for Medical Sciences, Little Rock, Arkansas, USA.; 3 Central Arkansas Veterans Healthcare System, Little Rock, Arkansas, USA.; 4 Department of Orthopedic Surgery, University of Arkansas for Medical Sciences, Little Rock, Arkansas, USA.

**Keywords:** Bone Biology, Mitochondria, Osteoclast/osteoblast biology, Osteoporosis

## Abstract

Altered mitochondria activity in osteoblasts and osteoclasts has been implicated in the loss of bone mass associated with aging and estrogen deficiency — the 2 most common causes of osteoporosis. However, the mechanisms that control mitochondrial metabolism in bone cells during health or disease remain unknown. The mitochondrial deacetylase sirtuin-3 (Sirt3) has been earlier implicated in age-related diseases. Here, we show that deletion of Sirt3 had no effect on the skeleton of young mice but attenuated the age-related loss of bone mass in both sexes. This effect was associated with impaired bone resorption. Osteoclast progenitors from aged Sirt3-null mice were able to differentiate into osteoclasts, though the differentiated cells exhibited impaired polykaryon formation and resorptive activity, as well as decreased oxidative phosphorylation and mitophagy. The Sirt3 inhibitor LC-0296 recapitulated the effects of Sirt3 deletion in osteoclast formation and mitochondrial function, and its administration to aging mice increased bone mass. Deletion of Sirt3 also attenuated the increase in bone resorption and loss of bone mass caused by estrogen deficiency. These findings suggest that Sirt3 inhibition and the resulting impairment of osteoclast mitochondrial function could be a novel therapeutic intervention for the 2 most important causes of osteoporosis.

## Graphical abstract

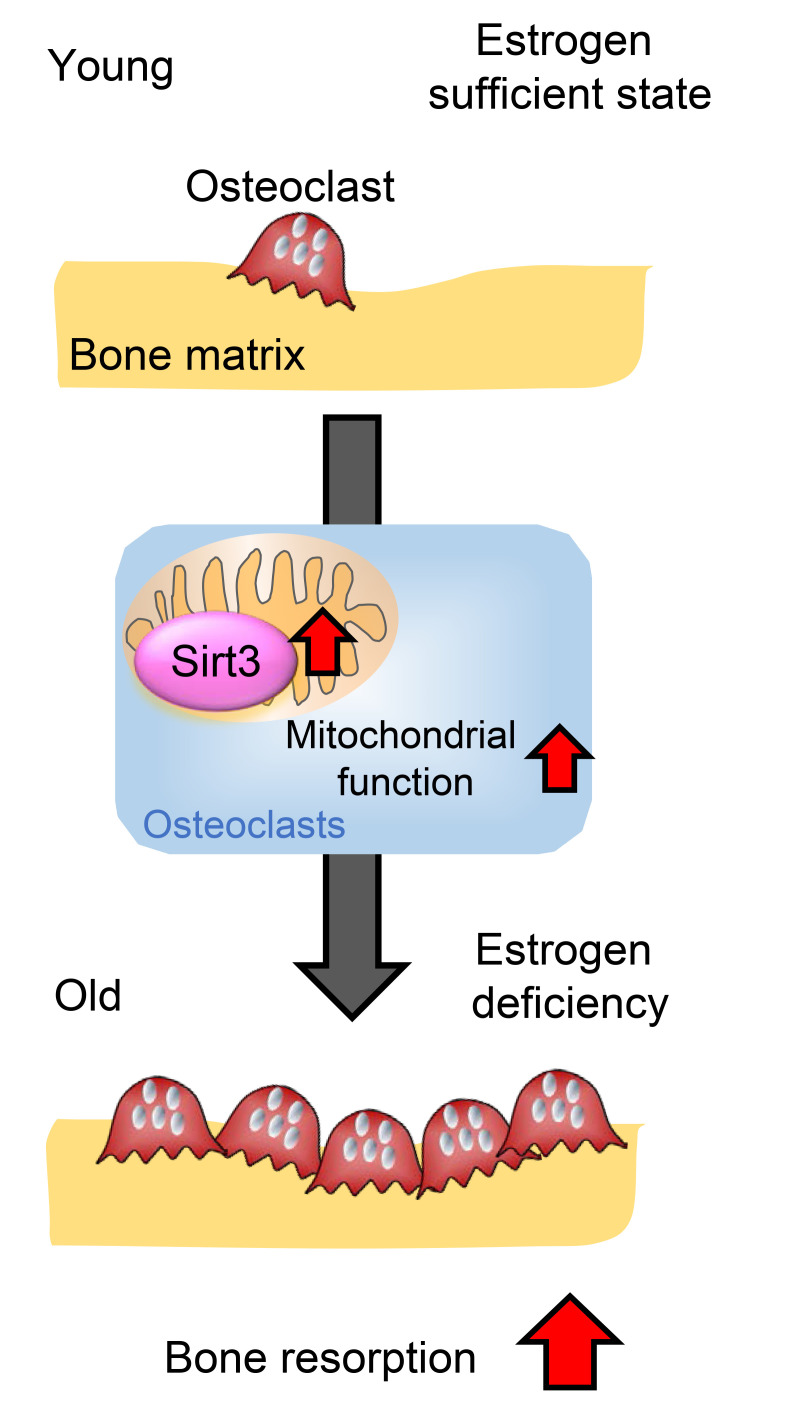

## Introduction

A balance between the function of bone-resorbing osteoclasts and bone-building osteoblasts is essential for bone homeostasis. Resorption of the mineralized bone matrix — a physiologic process essential for skeletal and mineral homeostasis — is the function of osteoclasts, multinucleated cells derived from myeloid precursors (1–4). During osteoclastogenesis, BM macrophages (BMMs) differentiate into tartrate-resistant acid phosphatase^+^ (TRAP^+^) preosteoclasts in response to the receptor activator of NF-κΒ (RANKL) (5). Mononuclear preosteoclasts then fuse with each other to form multinucleated mature osteoclasts. These highly specialized cells are uniquely capable of dissolving and digesting organic bone matrix (5). Most likely because of the high energy demands of these tasks, a distinct cellular feature of osteoclasts is the high abundance of mitochondria (6, 7).

The NAD^+^-dependent sirtuin-3 (Sirt3) is the primary mitochondrial protein deacetylase and plays a critical role in mitochondrial quality control, including mitochondrial biogenesis, mitochondrial dynamics, and mitophagy — all of which are affected in age-related metabolic diseases (8–11). Earlier work aiming to elucidate the role of Sirt3 in bone has produced conflicting results, with some studies suggesting that Sirt3 is critical for skeletal homeostasis (12, 13), while others found no physiological role of Sirt3 in bone (14, 15).

Sex steroid deficiency, aging, and inflammation cause an increase in osteoclast numbers and, thereby, bone loss (1–4). We and others have shown that RANKL-induced stimulation of mitochondrial function in osteoclasts is critical for osteoclastogenesis under physiologic conditions and contributes to loss of bone mass with estrogen deficiency (16–21). We have also shown that suppression of osteoclast formation by estrogens is associated with decreased mitochondria OxPhos and ATP production in early osteoclast progenitors (22). Furthermore, increased mitochondrial ROS levels in cells of the osteoblast lineage contributes to the loss of bone mass with age (23). Collectively, these findings strongly implicate altered mitochondrial metabolism in both osteoclasts and osteoblasts as a culprit of bone disease. However, the molecular mechanisms that control mitochondrial function in skeletal health and disease remain largely unknown. We show herein that deletion of Sirt3 or pharmacologic inhibition of Sirt3 in mice impairs osteoclast mitochondrial function and prevents the increased bone resorption and loss of bone mass caused by the 2 most important causes of osteoporosis in humans — aging and estrogen deficiency.

## Results

### The age-associated loss of bone mass is prevented in Sirt3-KO mice.

**Figure 1:**
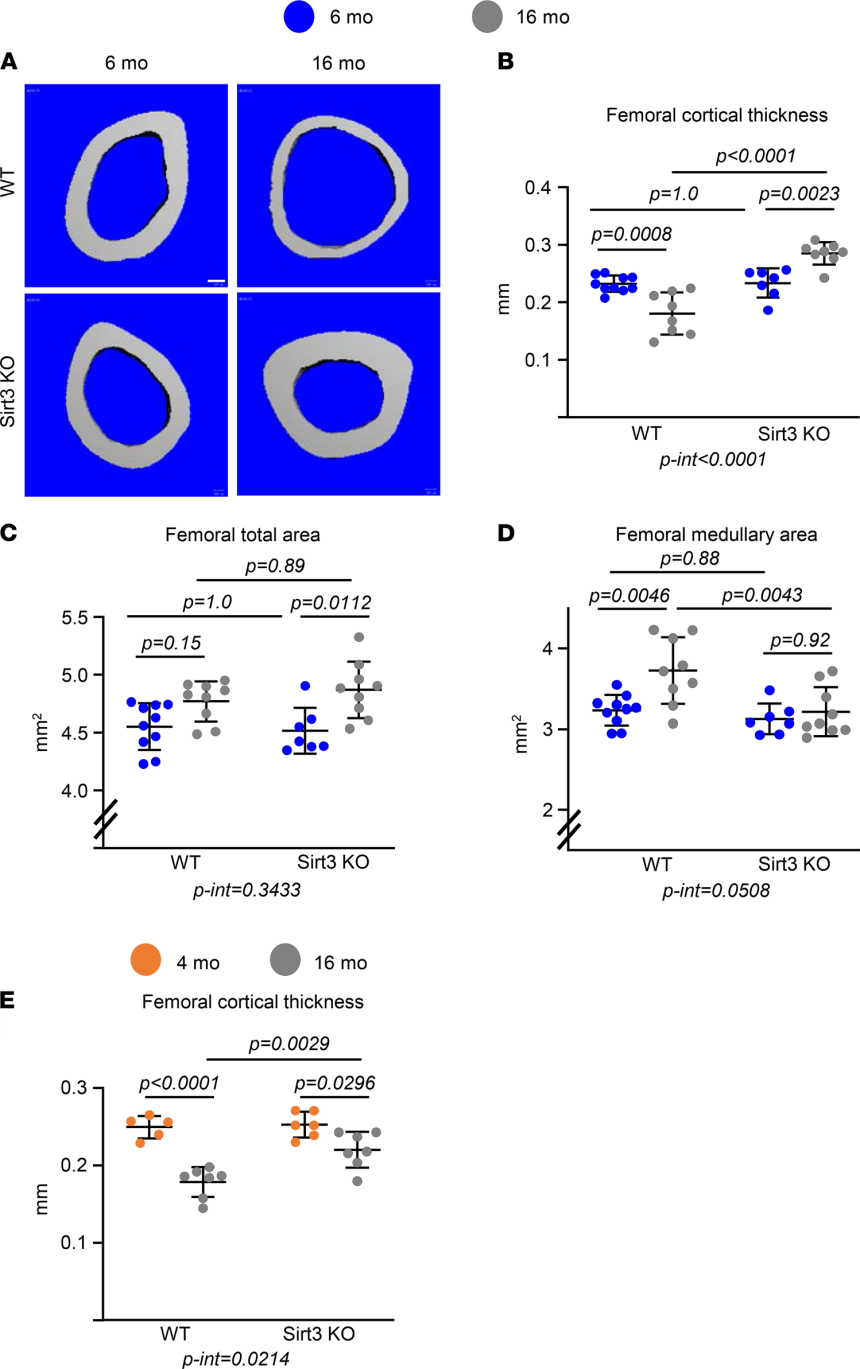
Deletion of Sirt3 prevents age-associated cortical bone loss. (**A**–**D**) Imaging and quantification of femoral bones from female Sirt3-KO mice and WT littermates by micro-CT after sacrifice (*n* = 7–10 animals/group). (**A**) Representative images of femoral cortical bone at midshaft. Scale bar: 100 μm. (**B**–**D**) Cortical thickness (**B**) and cortical perimeters (**C** and **D**) at the femoral midshaft. (**E**) Cortical thickness at the femoral midshaft of the femur from male Sirt3-KO mice and WT littermates by micro-CT (*n* = 5–7 animals/group). Data are presented as ± SD. *P* values were determined using 2-way ANOVA. Interaction terms generated by 2-way ANOVA are shown below each graph.

**Figure 2:**
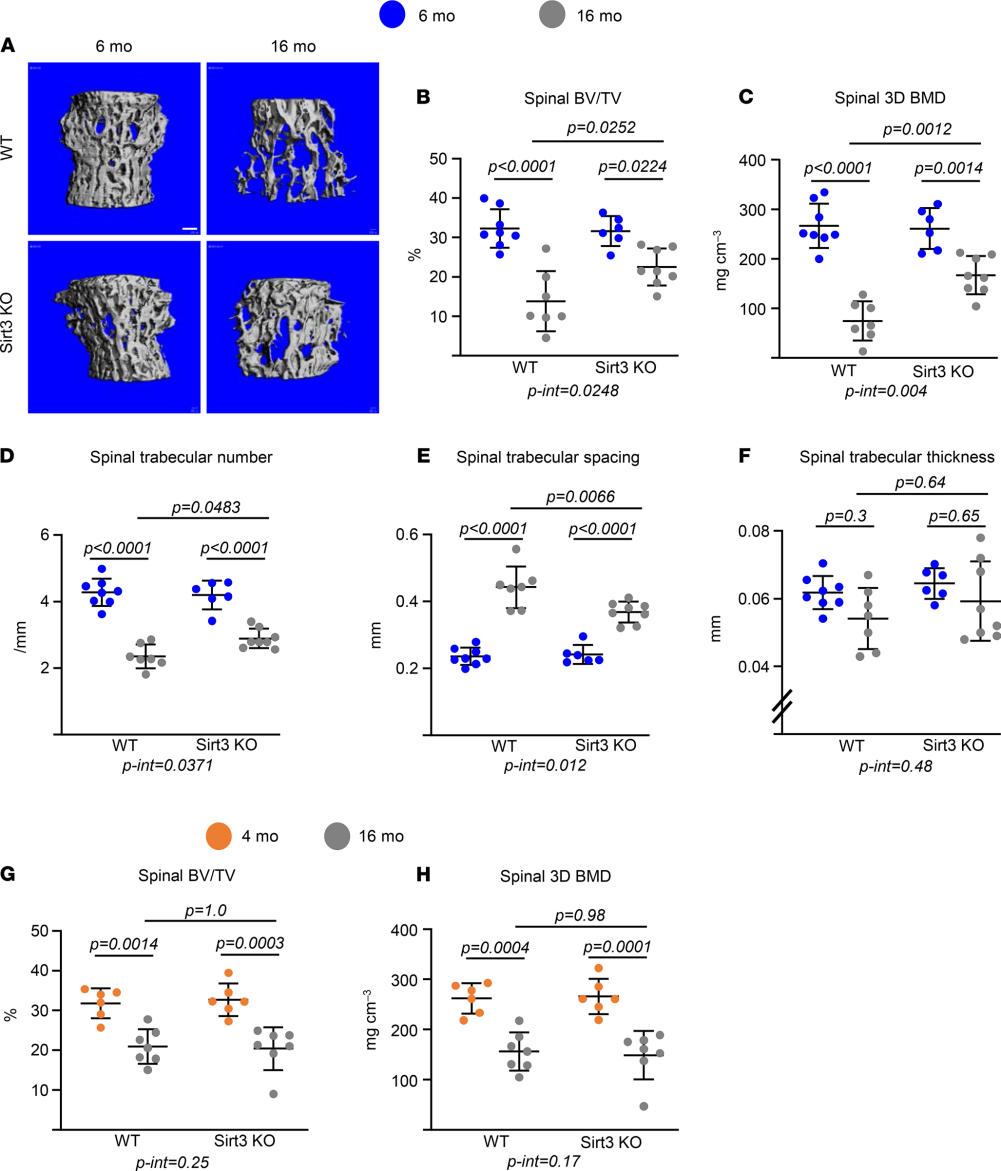
Deletion of Sirt3 attenuates age-associated trabecular bone loss. (**A**–**F**) Imaging and quantification of vertebral bones from female Sirt3-KO mice and WT littermates by micro-CT after sacrifice (*n* = 7–10 animals/group). Representative images of trabecular bone (**A**) and bone volume per tissue volume (BV/TV), bone mineral density (BMD), and microarchitecture of trabecular bone in L5 (**B**–**F**). Scale bar: 100 μm. (**G** and **H**) BV/TV and BMD from male Sirt3-KO mice and WT littermates in L5 bones by micro-CT (*n* = 5–7 animals/group). Data are presented as ± SD. *P* values were determined using 2-way ANOVA. Interaction terms generated by 2-way ANOVA are shown below each graph.

Mice heterozygous for the *Sirt3* mutant allele were intercrossed to produce homozygous KO mice. Cohorts of Sirt3-KO and littermate WT mice were aged up to 16 months. Consistent with previous studies (24), Sirt3-KO mice appeared normal at birth and were indistinguishable from WT mice with respect to size, body weight (data not shown), and femoral length during adulthood (Supplemental Figure 1; supplemental material available online with this article; https://doi.org/10.1172/jci.insight.146728DS1). As determined by micro-CT, 6-month-old female or 4-month-old male Sirt3-null mice exhibited no differences in cortical or trabecular bone mass (Figure 1, Figure 2, and Supplemental Figure 2) at the femur and spine compared with their age-matched controls. As expected, WT mice lost bone mass with age. Specifically, 16-month-old WT female mice had lower femoral cortical thickness compared with 6-month-old mice of the same genotype (Figure 1, A and B). The decrease in cortical thickness in the aged WT female mice was due to a disproportionate increase in medullary area as compared with the increase in total bone area (Figure 1, C and D). Male WT mice also exhibited a decrease in cortical thickness with age (Figure 1E), as previously described (25, 26). Cortical thickness in both female and male Sirt3-KO mice at 16 months of age was greater than WT controls of the same age (Figure 1, A, B, and E). More striking, 16-month-old Sirt3-null female mice had thicker cortical bone than 6-month-old mice of the same genotype (Figure 1, A and B). This was due to the lack of enlargement of the medullary cavity in the face of the expected enlargement of bone (total area) that occurs with age (Figure 1, C and D). The loss of cortical bone with age was also attenuated in Sirt3-KO males — but to a lesser degree than in females (Figure 1E).

In WT female mice, trabecular bone volume (bone volume per tissue volume [BV/TV]) and 3D bone mineral density (BMD) decreased between 6 and 16 months of age at both the spine (Figure 2, A–C) and the femur (Supplemental Figure 2, A–C). This change was associated with a decrease in trabecular number and an increase in trabecular spacing, while no changes were detected in trabecular thickness (Figure 2, D–F, and Supplemental Figure 2, D–F). Female Sirt3-null mice also lost trabecular bone at the spine with age (29%) but to a lesser extent than WT controls (57.2%) (Figure 2, A and B). This decrease was associated with an increase in trabecular number and a decrease in trabecular spacing (Figure 2, D and E). The loss of trabecular bone in the distal femur was also greatly attenuated in the Sirt3-null female mice (Supplemental Figure 2, A–C). In contrast to females, the deletion of Sirt3 had no effect on the age-related loss of trabecular bone mass of the femur or spine in male Sirt3-null mice (Figure 2, G and H, and Supplemental Figure 2, G and H). Taken together, these results indicate that Sirt3 contributes to the age-related bone loss in mice and that its contribution is greater in female than male mice and more pronounced in cortical than trabecular bone.

### Deletion of Sirt3 decreases resorption markers in vivo and osteoclast function in vitro.

**Figure 3:**
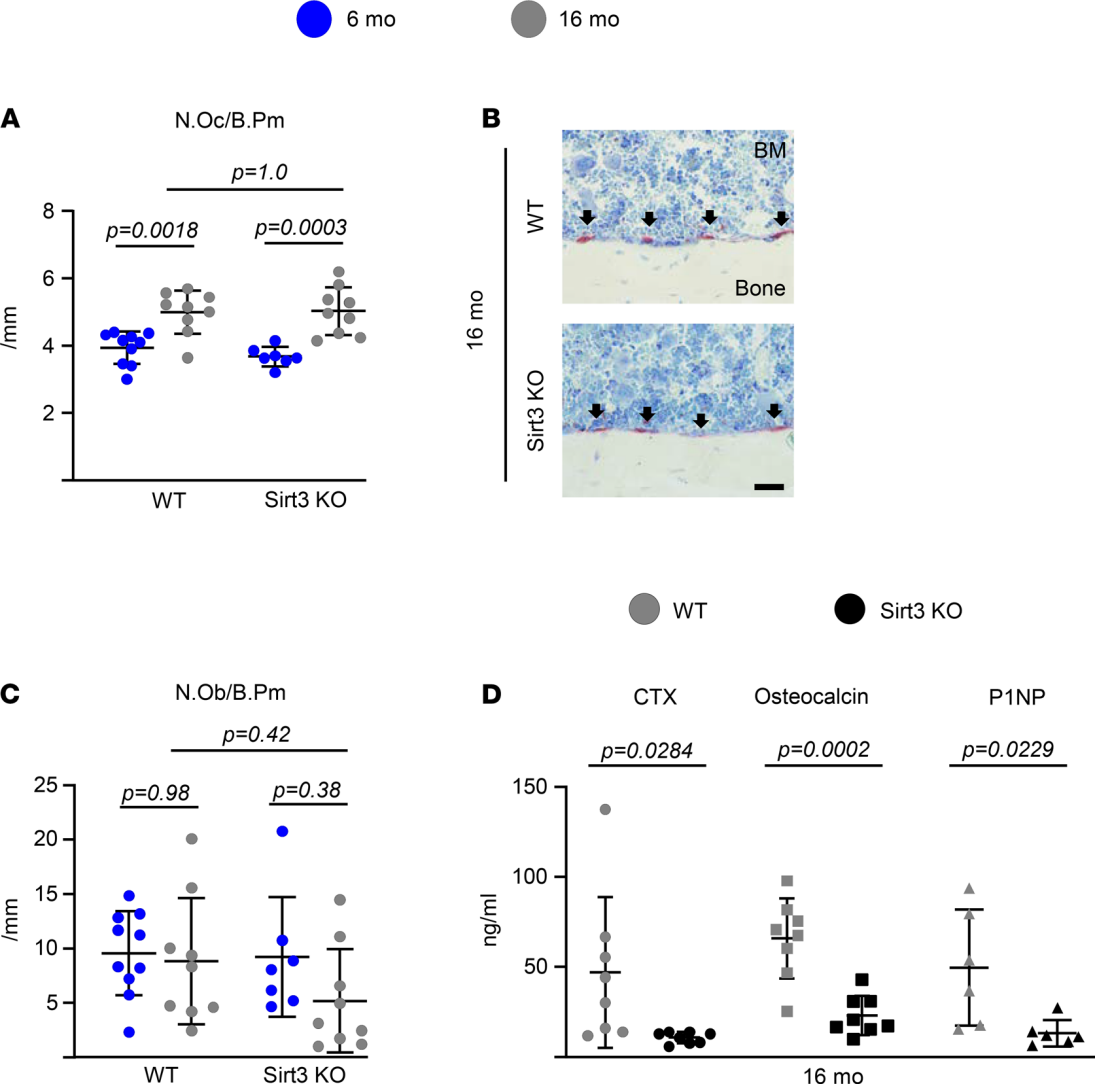
Deletion of Sirt3 decreases bone resorption in aged mice. (**A**–**C**) Number of osteoclast (N.Oc/B.Pm) (**A**) and osteoblast (N.Ob/B.Pm) per endocortical bone surface (**C**), and representative photomicrographs of nondecalcified femur sections stained for TRAPase activity (red) (**B**) from 16-month-old female Sirt3-KO mice and WT littermates (*n* = 9 animals/group). Scale bar: 100 μm. (**D**) Serum concentration of a collagen degradation product (CTx), osteocalcin, and N-terminal propeptide of type I procollagen (P1NP) in 16-month-old female Sirt3-KO mice and WT littermates by ELISA (*n* = 6–8 animals/group). Data are presented as ± SD. *P* values were determined using (**A** and **C**) 2-way ANOVA or (**D**) Student’s *t* test.

Age-related bone loss is associated with decreased osteoblasts and increased osteoclast numbers. To gain insight into the cellular mechanisms of the protective effect of Sirt3 deletion on skeletal aging, we first performed histologic analysis to evaluate osteoclast and osteoblast numbers at the endocortical surface of the femur. Sixteen-month-old Sirt3-KO female mice showed no differences in osteoclast or osteoblast number (Figure 3, A–C). Strikingly, however, the serum levels of C-terminal telopeptide of type 1 collagen (CTx) — osteocalcin and N-terminal propeptide of type I procollagen (P1NP), markers of bone resorption and bone formation, respectively — were dramatically lower in Sirt3-KO mice (Figure 3D). This finding suggests that the high bone mass of these mice is, at least in part, due to a decrease in osteoclastic bone resorption and the resorption-driven bone turnover.

**Figure 4:**
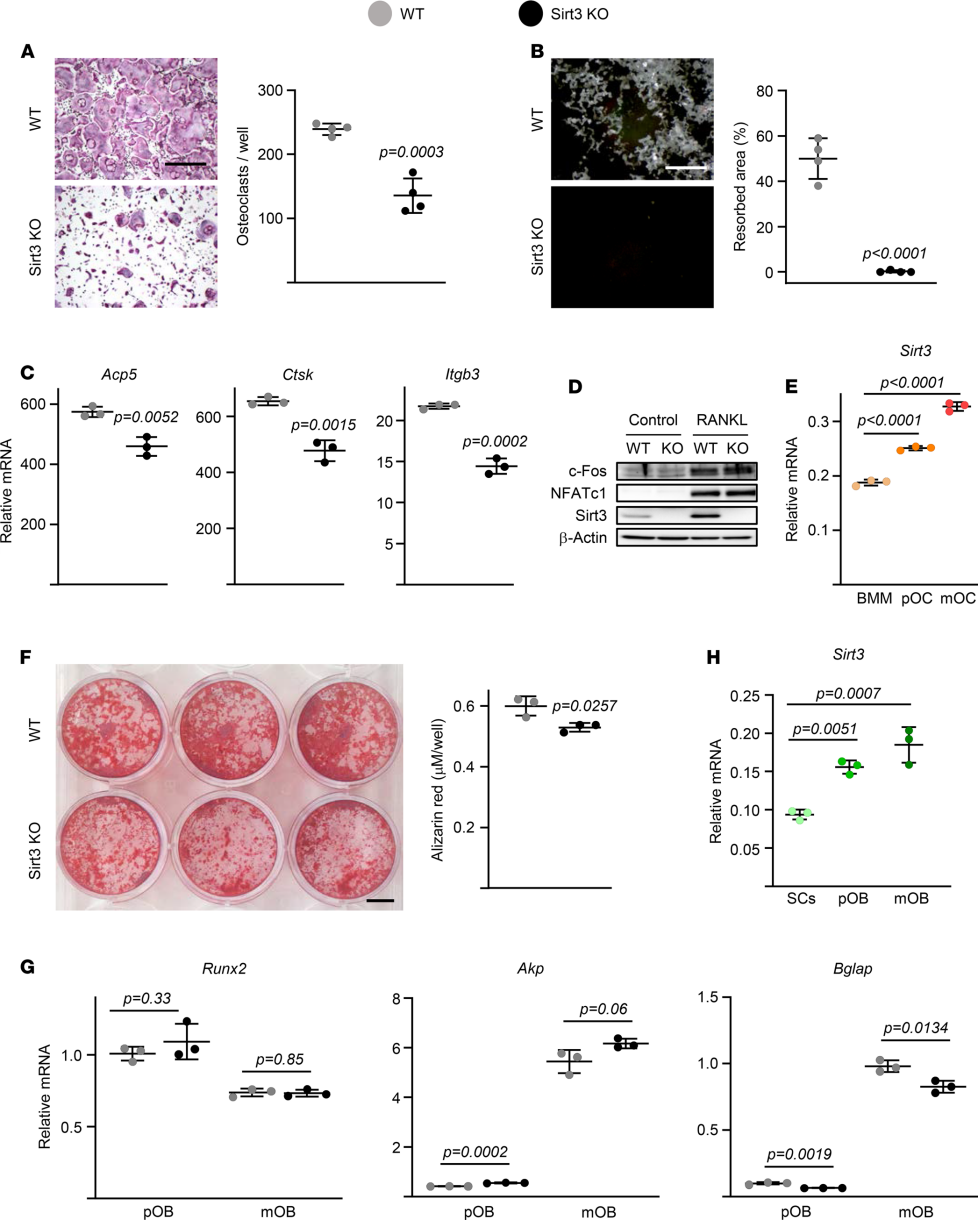
Deletion of Sirt3 decreases osteoclast function in aged mice. (**A**–**C**) BMMs were isolated from 16-month-old female Sirt3-KO mice and WT littermates and cultured with M-CSF (30 ng/mL) and RANKL (30 ng/mL) for 5 days (**A** and **B**) or 2 days (**C**). (**A**) Representative pictures (left) and number (right) of TRAP^+^ multinucleated osteoclasts generated from BMMs (quadruplicates of pooled cultures). Scale bar: 500 μm. (**B**) Representative pictures (left) and resorbed areas (right) of Von Kossa–stained bone biomaterial surface (quadruplicate cultures). Scale bar: 500 μm. The resorbed areas appear white, and the unresorbed mineralized surface appears black. (**C**) Osteoclast marker levels in mRNA of cultured osteoclasts measured by qPCR (triplicate cultures). (**D**) Protein levels by Western blot in BMM cell cultures (triplicate cultures). (**E**) BMMs were isolated from 6-month-old C57BL/6 WT mice and cultured with M-CSF (30 ng/mL, BMM) or with M-CSF and RANKL (30 ng/mL) for 2 days (pOC) or 5 days (mOC). *Sirt3* levels in mRNA during osteoclastogenesis by qPCR assay (triplicate cultures). (**F**–**H**) BM stromal cells were isolated from 16-month-old female Sirt3 knockout mice and WT littermate controls (**F** and **G**) or 6-month-old C57BL/6 WT mice (**H**) cultured with ascorbate (50 mg/mL; SCs) or ascorbate and β-glycerophosphate (10 mM) for 3 days (pOB) or 14 days (mOB). (**F**) Representative pictures (left) and quantification (right) of Alizarin Red staining in mOB (triplicates of pooled cultures). Scale bar: 1 cm. (**G** and **H**) Osteoblast marker and *Sirt3* levels in mRNA of SCs, pOB, and mOB measured by qPCR (triplicate cultures). pOC, preosteoclasts; pOB, osteoblasts; mOC, mature osteoclasts; mOB; mature osteoblasts. Data are presented as ± SD. *P* values were determined using Student’s *t* test (**A**–**C**, **F**, and **G**) or 1-way ANOVA (**E** and **H**). All measures were performed in cultured BMMs or stromal cells pooled from 4–5 mice/group.

To probe into the mechanism of the decreased resorption in the Sirt3-null mice in the face of unchanged osteoclast numbers, we examined osteoclast function. Cultures of BMMs from aged mice lacking Sirt3 formed smaller osteoclasts than those from WT mice (Figure 4A). Moreover, the area resorbed by Sirt3-deficient osteoclasts was greatly diminished in these cultures (Figure 4B). The expression of late/terminal osteoclast differentiation markers was decreased in cultured osteoclasts from aged Sirt3-KO mice (Figure 4C). Protein levels of factors known to control early osteoclast differentiation, such as c-Fos and NFATc1 (27), were not affected by Sirt3 deletion (Figure 4D). Nonetheless, both protein and mRNA levels of *Sirt3* were greatly upregulated during the process of osteoclastogenesis, as shown in macrophage cultures from WT mice stimulated with RANKL (Figure 4, D and E). This result is consistent with the requirement of Sirt3 for optimal mitochondrial biogenesis and function in many different cell types.

BM-derived stromal cells from aged Sirt3-KO mice cultured under osteogenic conditions exhibited a mild decrease in osteoblast differentiation (Figure 4F). The mRNA levels of the early osteoblastogenesis markers *Runx2* and *Akp* were unaffected by the Sirt3 deletion. However, osteocalcin was decreased in cultured stromal cells from aged Sirt3-KO mice (Figure 4G). In cultures derived from WT mice, the mRNA levels of *Sirt3* were upregulated during osteoblastogenesis (Figure 4H). Taken together, these results suggest that the increase in bone resorption associated with aging depends, at least in part, on direct effects of Sirt3 on osteoclasts.

### Deletion of Sirt3 decreases mitochondrial respiration and mitophagy in osteoclasts.

**Figure 5:**
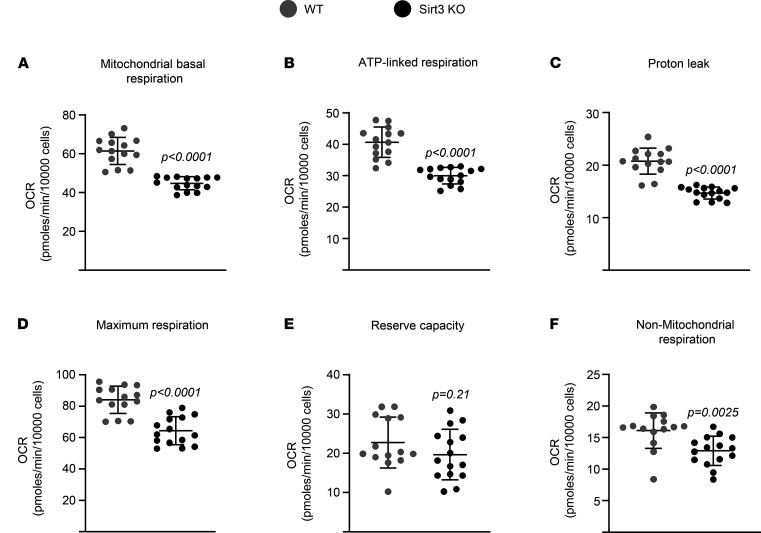
Deletion of Sirt3 attenuates respiration in osteoclasts of aged mice. (**A**–**F**) BMMs were isolated from 16-month-old female Sirt3-KO mice and WT littermate controls and cultured with M-CSF (30 ng/mL) and RANKL (30 ng/mL) for 3 days. Different fractions of mitochondrial and nonmitochondrial respirations per cell, in osteoclasts, measured by Seahorse (*n* = 14–15 wells/group). Data are presented as ± SD. *P* values were determined using Student’s *t* test. All measures were performed in cultured BMMs pooled from 4–5 mice/group.

We next examined whether Sirt3 alters mitochondrial function in osteoclasts by performing extracellular flux analysis. Mitochondrial respiration was significantly diminished in osteoclasts from aged Sirt3-KO mice (Figure 5A). Deletion of Sirt3 also decreased ATP-linked respiration, proton leak, maximum respiration (maximal electron transport chain activity), and nonmitochondrial respiration but had no impact on reserve respiratory capacity — the difference between the maximum oxygen consumption rate (OCR) and basal respiratory OCR that provides cells flexibility during increased energy demands (Figure 5, B–F). However, Sirt3 lacking osteoclasts from young mice exhibited no difference in mitochondrial respiration compared with the cells from age-matched controls (Supplemental Figure 3B). Consistent with this, the inhibitory effect of Sirt3 deletion on osteoclast differentiation seen in cells from old mice (16-month-old mice; –103.5% ± 14.18%; Figure 4A) was mild in cells from young mice (–42.75% ± 5.50%; Supplemental Figure 3A). These results suggest that Sirt3 contributes to the increase in osteoclast function and bone resorption primarily during skeletal aging.

**Figure 6:**
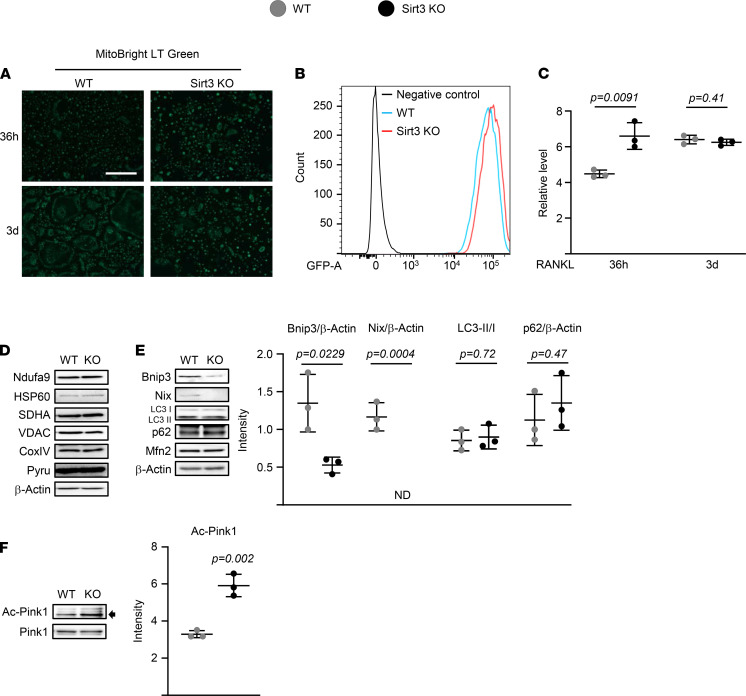
Mitophagy is the dominant Sirt3 mechanism contributing to osteoclast function. (**A**–**F**) BMMs were isolated from 16-month-old female Sirt3 knockout mice and WT littermate controls and cultured with M-CSF (30 ng/mL) and RANKL (30 ng/mL) for indicated times (**A** and **C**), 36 hours (**B**), or 3 days (**D**–**F**). (**A**) Representative pictures of MitoBright Green staining in cultures by fluorescence imaging. (**B** and **C**) Representative analysis (**B**) and quantification (**C**) of MitoBright Green signals by FACS (triplicate cultures). Scale bar: 500 μm. (**D**–**F**) Representative mitochondrial protein levels by Western blot and expression levels as the indicated ratio (triplicate cultures). Data are presented as ± SD. *P* values were determined using Student’s *t* test. All measures were performed in cultured BMMs pooled from 4–5 mice/group.

We also examined mitochondrial content using MitoBright Green staining and expression levels of 2 genes encoded by the mitochondria genome, *ND2* and *Cytb* (normalized to genomic DNA content). Both fluorescence imaging and flow cytometry analysis of mitochondria of differentiated osteoclasts revealed no differences in MitoBright Green staining between the 2 genotypes (Figure 6, A–C); in fact, the signals were slightly higher in osteoclast progenitors from Sirt3-KO mice. Likewise, Sirt3 deletion had no effect on the expression of mitochondrial DNA–encoded genes or on mRNA levels of *Pgc1b* or *Tfam* — 2 transcription factors that regulate mitochondrial biogenesis (28) (Supplemental Figure 4). The expression of several proteins representative of mitochondria, such as NAD dehydrogenase (NDUFA9; complex I), succinate dehydrogenase complex subunit A (SDHA; complex II), cytochrome C oxidase IV (COX IV; complex V), pyruvate dehydrogenase (PDH, inner mitochondrial membrane), heat-shock protein 60 (HSP60; mitochondrial chaperonin), and voltage-dependent anion channel (VDAC, outer mitochondria membrane) (29–34) were also unaffected by Sirt3 deletion (Figure 6D). These results suggest that Sirt3 deletion inhibits mitochondrial respiration independently of changes in mitochondrial content.

We next examined whether Sirt3 deletion altered the abundance of proteins involved in mitochondria dynamics and mitophagy. Mitofusin2 mediates the tethering of adjacent mitochondria, as well as the tethering of mitochondria to the endoplasmic reticulum (ER), and promotes osteoclast differentiation (35). Sirt3 deletion had no impact on the protein levels of Mitofusin2 in BMM stimulated with RANKL (Figure 6E). Sirt3 activates mitophagy via Pink1 and increases expression of Bnip3 and Nix (36–38). The protein levels of the macroautophagy markers LC3 and p62 were unaffected by Sirt3 deletion. However, Bnip3 and Nix were reduced in cultured osteoclasts from aged Sirt3-KO mice (Figure 6E). Interestingly, osteoclasts lacking Sirt3 had increased Pink1 acetylation but produced no changes in total protein levels (Figure 6F). These results indicate that Sirt3-mediated Pink1 deacetylation might regulate mitophagy in osteoclasts, which in turn promotes osteoclast differentiation. Indeed, Sirt3 induces mitophagy via direct deacetylation of Pink1 in primary mouse cardiac microvascular endothelial cells (39).

### The Sirt3 inhibitor LC-0296 increases bone mass in aging mice.

**Figure 7:**
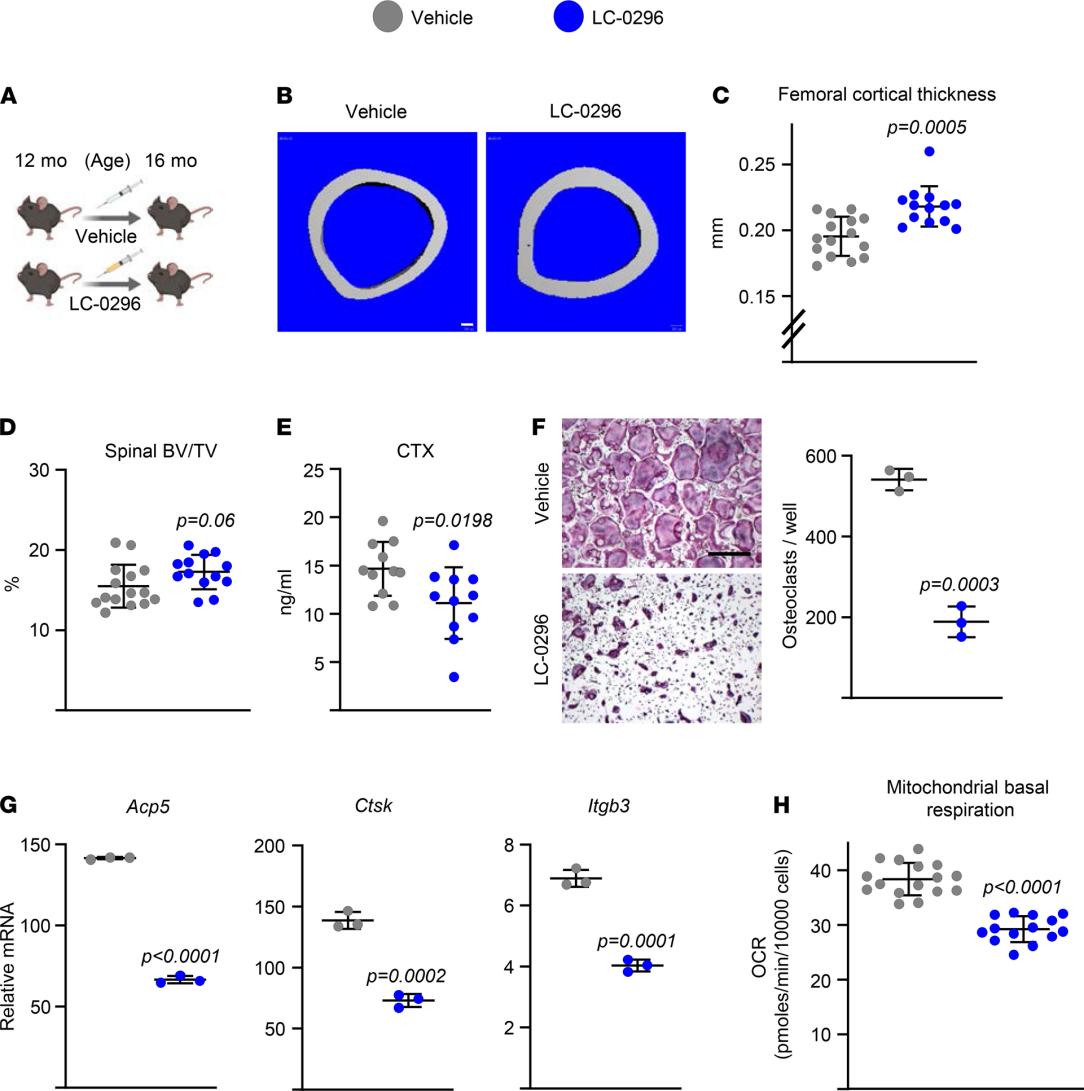
Administration of LC-0296 increases bone mass in aging mice by attenuating bone resorption. (**A**) Schedule of LC-0296 administration (5 μg/g body weight, 100 μL/each i.p. injection) to 12-month-old female C57BL/6 mice. (**B** and **C**) Representative images (**B**) and cortical thickness (**C**) of femoral cortical bone at midshaft. Scale bar: 100 μm. (**D**) BV/TV of trabecular bone in L5 measured by micro-CT (*n* = 13–15 animals/group). (**E**) Serum CTx by ELISA (*n* = 11 animals/group). (**F**–**H**) Osteoclasts developed in cultures of BMMs from 16-month-old female C57BL/6 mice with M-CSF (30 ng/mL) and RANKL (30 ng/mL) for 5 days (**F**) or 3 days (**G** and **H**), in the presence or absence of LC-0296 (10 nM). (**F**) Representative pictures (left) and number (right) of TRAP^+^ multinucleated osteoclasts (triplicate cultures). Scale bar: 500 μm. (**G**) Osteoclast marker levels in mRNA of cultured osteoclasts measured by qPCR (triplicate cultures). (**H**) Mitochondrial respiration per cell, measured by Seahorse (*n* = 14–16 wells/group). Data are presented as ± SD. *P* values determined using Student’s *t* test. All in vitro assays were performed in cultured BMMs pooled from 3 mice.

To obtain independent confirmation for a role of Sirt3 in skeletal aging, we examined the effects of LC-0296, a synthetic small molecule antagonist of Sirt3. This compound inhibits Sirt3 enzymatic activity with about 20-fold greater efficacy than other Sirtuins (40). Administration of LC-0296 to 12-month-old female WT C57BL/6 mice for 4 months (Figure 7A) had no impact on body weight and caused no toxicity, as indicated by the absence of inflammatory cell infiltration or accumulation of damaged cells in heart, kidney, liver, and lung (Supplemental Figure 5). LC-0296 treatment increased femoral cortical thickness (Figure 7, B and C). A modest increase in trabecular bone mass was also noted in vertebrae (Figure 7D). Consistent with these changes, serum CTx levels decreased in LC-0296–treated mice (Figure 7E). These results indicate that LC-0296 mimics, at least in part, the effects of Sirt3 deletion and increases bone mass in aging mice. Addition of LC-0296 to cultures of osteoclast progenitors from 16-month-old female C57BL/6 mice decreased osteoclast formation (Figure 7F) and expression of late/terminal osteoclast differentiation markers (Figure 7G). Most osteoclasts formed in the presence of LC-0296 were much smaller (Figure 7F), reminiscent of the osteoclasts from aged Sirt3-KO mice. Additionally, LC-0296 decreased mitochondrial respiration in osteoclasts (Figure 7H and Supplemental Figure 6).

### Deletion of Sirt3 protects against ovariectomy-induced cortical but not trabecular bone loss.

**Figure 8:**
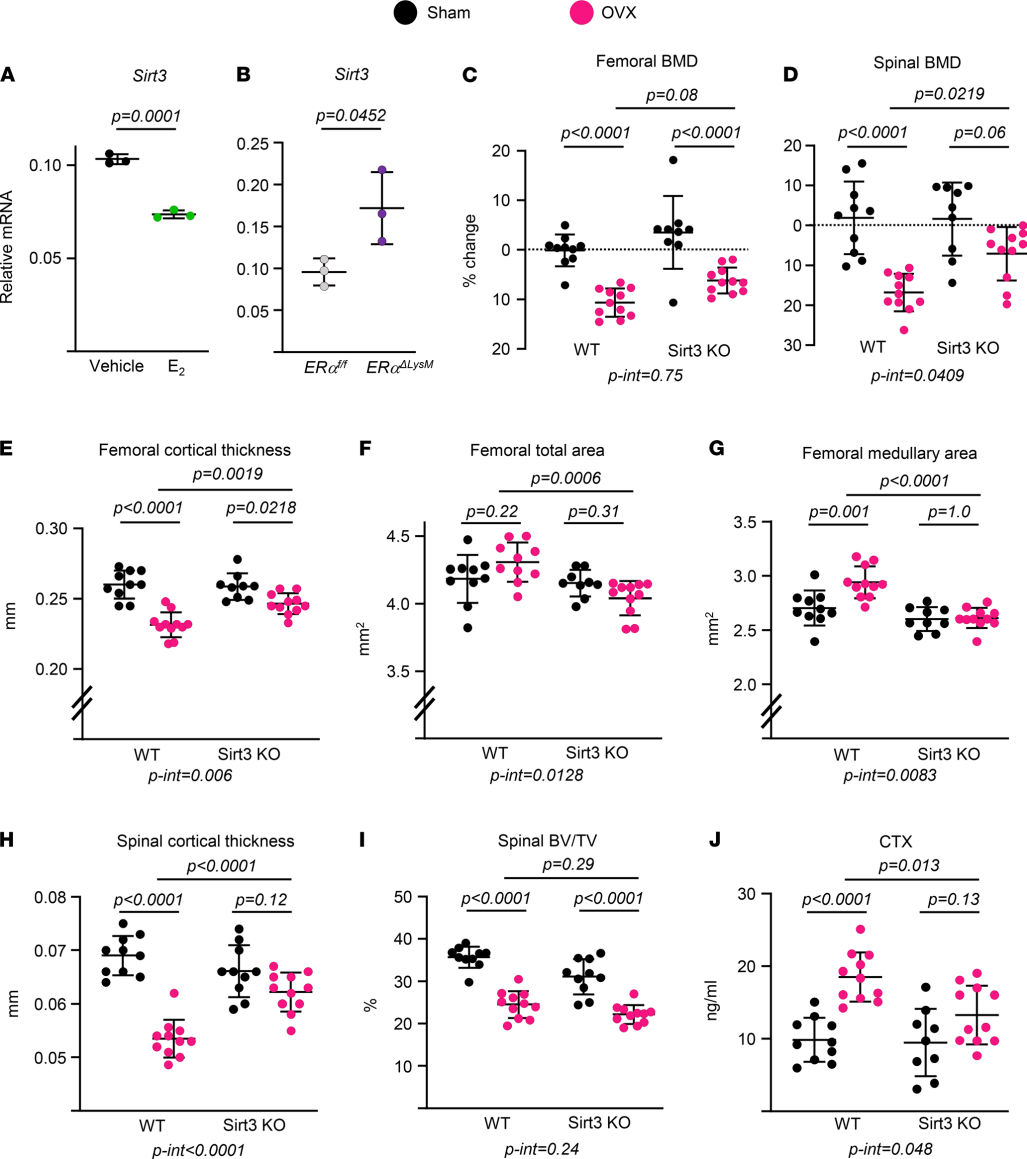
Deletion of Sirt3 attenuates ovariectomy-induced bone loss. (**A** and **B**) *Sirt3* mRNA by qPCR in BMMs isolated from 6-month-old female C57BL/6 mice (**A**) or 3-month-old females of the indicated genotype (**B**) and cultured with M-CSF (30 ng/mL) and RANKL (30 ng/mL) for 2 days in the presence or absence of E_2_ (1 × 10^–8^M) (triplicate cultures). (**C**–**J**) Five-month-old female Sirt3-KO mice and WT littermates were sham operated or ovariectomized (OVX) for 6 weeks (*n* = 9–11 animals/group). (**C** and **D**) Percent change in BMD by DXA 1 day before surgery and before sacrifice. (**E**–**H**) Cortical thickness and areas at the femoral midshaft (**E**–**G**) and L5 bones measured by micro-CT (**H**) (*n* = 9–11 animals/group). (**I**) BV/TV of trabecular bone in L5 by micro-CT (*n* = 9–11 animals/group). (**J**) Serum CTx concentration measured by ELISA (*n* = 9–11 animals/group). Data are presented as ± SD. *P* values were determined using Student’s *t* test (**A** and **B**) or 2-way ANOVA (**C**–**J**). Interaction terms generated by 2-way ANOVA are shown below each graph. All in vitro assays were performed in cultured BMMs pooled from 3–4 mice/genotype.

A decline of estrogen levels at menopause or following ovariectomy (OVX) causes bone loss in women and mice (2, 41). This loss occurs at both cancellous and cortical sites and is associated with high bone remodeling, as evidenced by increased numbers of both osteoclasts and osteoblasts (42). We have previously shown that 17β-estradiol (E2) inhibits mitochondrial function in osteoclast progenitors (22). Based on this background, we searched for and found that E2 decreased the mRNA levels of *Sirt3* in WT BMMs cultured with RANKL (Figure 8A). In contrast, BMMs from mice with conditional deletion of estrogen receptor α (ERα) in cells of the myeloid lineage (22, 43) had higher *Sirt3* mRNA levels (Figure 8B), suggesting that Sirt3 might be involved in the actions of estrogens in osteoclasts. To investigate this possibility, we performed OVX on Sirt3-KO mice and WT littermate controls at 5 months of age and examined the impact of estrogen deficiency on bone mass 6 weeks later. OVX caused a decrease of BMD in the femur and spine of WT and Sirt3-null mice, as determined by Dual-energy X-ray absorptiometry (DXA; Figure 8, C and D). Nonetheless, the OVX-induced loss of BMD was attenuated in Sirt3-null mice. OVX decreased cortical bone thickness in the femur of control mice (Figure 8E) due to an increase in the medullary area, while total area was not affected (Figure 8, F and G). Notably, the effects of OVX on cortical bone were greatly attenuated in Sirt3-KO mice (Figure 8, E and G). Similarly, cortical thickness in the vertebra was reduced with OVX in control mice but not in Sirt3-KO mice (Figure 8H). Two-way ANOVA showed a significant difference between genotypes in the magnitude of the bone loss at both sites (femur: p-int = 0.006 and spine: p-int < 0.0001). In contrast, the loss of trabecular bone and changes in microarchitecture with OVX were indistinguishable between control and Sirt3-KO mice in both vertebrae and femur (Figure 8I, Supplemental Figure 7, and Supplemental Figure 8). Finally, the OVX-induced increase in bone resorption, as determined by serum CTx levels, was attenuated by Sirt3 deletion (Figure 8J). We found no differences in bone formation parameters between the 2 genotypes in OVX experiments (Supplemental Figure 9), suggesting that the attenuation of OVX-induced bone loss in Sirt3-null mice is due to a decrease in bone resorption, instead of an increase in bone formation.

## Discussion

The increase of fractures associated with the loss of bone mass with old age or sex steroid deficiency represents a major clinical problem for both women and men (1, 25, 26, 44, 45). Numerous descriptive studies have demonstrated dysregulation of mitochondrial function in age-related diseases, but it is largely unknown whether altered mitochondrial function in skeletal cells plays a role in the decrease of bone mass in the elderly. Herein, we provide evidence in mice that the mitochondrial protein deacetylase Sirt3 is required for the full manifestation of the effects of aging and OVX on bone resorption; and its adverse effects, most likely, result from enhanced mitochondrial function in osteoclasts.

Deletion of Sirt3 decreased bone resorption but had no effect on osteoclast number in trabecular or endocortical bone surfaces. Similar findings have been reported in mice lacking other proteins important for mitochondria. For example, global deletion of PGC1β, a critical transcription factor for mitochondrial oxidative energy metabolism, decreases mitochondrial biogenesis in osteoclasts and increases bone mass (16). Serum levels of CTx are diminished in PGC1β-KO mice, but the number of osteoclasts in bone is indistinguishable from that of control mice. Likewise, mice with conditional deletion of PGC1β in myeloid cells have normal osteoclast number in bone but display high bone mass compared with control littermates (46). These effects of PGC1β are reminiscent of the effect of Sirt3 and support the contention that mitochondria metabolism promotes osteoclast differentiation and bone resorption.

Sirt3 promotes mitochondria metabolism by deacetylating several target proteins. We found that the acetylation of Pink1 was increased and the levels of Bnip3 and Nix were decreased in osteoclasts from aged Sirt3-KO mice, suggesting that Sirt3 promotes osteoclast formation and function, in part, by stimulating mitophagy. Mitochondrial protein turnover by mitophagy is critical for the maintenance of healthy functional mitochondria. Pink1 and the ubiquitin ligase Parkin normally work together in the same pathway to govern mitochondrial quality control, and loss of function of the 2 proteins are involved in many cases of early-onset familial Parkinson’s disease (47). Sirt3 activates Pink1 to promote the ubiquitination of mitochondrial outer membrane proteins (48). Mitochondria dysfunction frequently mediates the cellular impairment caused by disruption of autophagy. Interestingly, studies with mice lacking critical proteins for autophagy in osteoclasts have revealed that autophagy promotes bone resorption and mediates the loss of bone mass caused by estrogen deficiency (49). Overall, these findings support the notion that the effects of Sirt3 in osteoclasts are due, at least in part, to mitophagy. Nonetheless, a broader analysis of lysine acetylome using proteomics will be required to identify the range of target proteins and mitochondria pathways that are responsible for the effects of Sirt3 during osteoclast development.

We have previously reported that the increase in osteoclast number with aging is associated with an increase in RANKL expression by osteocytes; this source of RANKL is indispensable for the age-related loss of bone at endocortical and intracortical surfaces (50). Several studies have shown that RANKL promotes mitochondria activity in osteoclast progenitor cells via multiple mechanisms, including increased mitochondria biogenesis and stimulation of OxPhos (16–21). We found here that RANKL increases the mRNA and protein levels of *Sirt3*, which in turn stimulates osteoclast formation and bone resorption, most likely by promoting mitochondria metabolism. In the absence of Sirt3, mitochondrial function in osteoclasts declines with age, resulting in osteoclasts with reduced function. This preserves bone mass in mice. Curiously, the skeletal effects of Sirt3 are relevant under stress but not homeostatic conditions. This contention is supported by findings that transgenic mice overexpressing Sirt3 exhibit cortical and trabecular bone loss at 13 months of age but not at 6 months of age (51). Likewise, much of the current knowledge on the functions of Sirt3 in tissues other than bone was obtained from studies with Sirt3-KO mice under pathological conditions. Specifically, in cells such as neurons, cardiomyocytes, and hepatocytes, Sirt3 exerts a protective role against inflammation, oxidative stress, and old age most likely by promoting deacetylation of its target proteins (8–11, 52). While the reasons for the different actions of Sirt3 under homeostatic and disease conditions remain unclear, recent proteomic studies have shed some light on the mechanisms via which Sirt3 exerts different roles in healthy versus stressed mitochondria (10). This study indicates that lower mitochondria matrix pH conditions due to mitochondrial membrane depolarization causes Sirt3 to change protein substrates and deacetylate proteins to promote oxidative metabolism and restore the proton gradient. These findings are in line with our results showing that Sirt3 promotes oxidative phosphorylation in osteoclastic cells from old mice. Furthermore, previous studies have shown that macrophages from old humans (≥65 years old) or mice exhibit mitochondria dysfunction (53). Together, these findings suggest that age-related changes in mitochondria of osteoclast lineage cells trigger the requirement for Sirt3 to maintain osteoclast formation and resorptive activities.

Estrogens inhibit osteoclast formation and protect against bone loss via direct actions on cells of the osteoclast lineage and indirectly via actions on cells of the mesenchymal lineage and perhaps other cell types, as well as several molecular mechanisms (1, 23, 43, 45). We have shown that mitochondrial H_2_O_2_ in osteoclasts plays a role in the loss of bone with estrogen deficiency (23). Furthermore, results from a recent in vitro study of ours have suggested that direct effects on osteoclast mitochondria mediate the antiosteoclastogenic actions of estrogens (22). For reasons that remain unclear, some mechanisms of estrogen action predominantly influence cortical bone, while others influence predominately trabecular bone. Here, we found that E2 decreases the expression of *Sirt3* in osteoclast progenitors and that the increase in bone resorption and loss of cortical, but not trabecular, bone mass with estrogen deficiency is attenuated in mice lacking Sirt3. Future studies are needed to further dissect the pathways via which estrogens directly and indirectly impact osteoclast mitochondria and why these predominate in distinct bone compartments. While our results point to a decrease in bone resorption as the culprit of the increased bone mass in aged or OVX Sirt3-KO mice, a disproportionate effect on bone resorption could mask an effect of Sirt3 on bone formation. Indeed, BM-derived osteoblast progenitors lacking Sirt3 exhibit a small decrease in osteoblastogenesis, and the serum levels of osteocalcin and P1NP were diminished in aged Sirt3-KO mice. Nevertheless, further genetic studies will be required to elucidate whether Sirt3 in osteoclast, osteoblast, or other cell types affect age- or estrogen deficiency–related bone loss.

Sirt3 protects against the loss of cortical bone with age in females but has a much milder effects in males, suggesting sex-related differences of Sirt3 actions in bone. During our previous studies aimed at characterizing skeletal aging in mice, we noticed that female and male C57BL/6 mice exhibit different features of bone aging (26). For example, the increase in intracortical bone remodeling is of greater magnitude in aged female than male mice. Others have also noted osteoclast related differences between males and females. Specifically, female mice have more osteoclasts and a lower bone mass in the trabecular compartment than males at any given age (54), and cultures of BMMs from female mice form more osteoclasts than those from male mice (55). In addition, Ballard et al. have reported that female but not male mice lacking mitochondrial fission protein Mitofusin2 in myeloid cells are protected from bone loss with age and from RANKL-induced osteolysis (35). The authors noted that this might be a threshold effect for mitochondrial function on osteoclast differentiation and that females have more sensitivity to Mitofusin2 loss than males. Along with this earlier evidence, our findings here support the notion that the mechanisms of skeletal aging are, in part, different between males and females and that sexual dimorphic responses of osteoclasts are the likely reason. In addition, our findings suggest that osteoclast mitochondria and Sirt3 might underlie the sex-related differences in mechanisms in these cells. Evidently, in humans, the sex-related differences in skeletal aging are attributed, in part, to hormonal changes at the menopause. Thus, in women, it is very difficult to dissect effects of aging from the effects of estrogen loss. In contrast, female mice maintain functional levels of estrogen until old age (23) and, thereby, represent a useful model to discern mechanisms of aging from mechanisms of estrogen deficiency, thus elucidating sex dimorphic effects on age-related bone loss.

We found no changes of bone mass in 6-month-old Sirt3-KO female or male mice, in line with earlier reports by Ho et al. and Busse et al. (15, 51). As in bone, Sirt3 has no effect under unstressed conditions in several other tissues (56–58). In contrast to the lack of an effect of Sirt3 deletion on bone mass, Gao et al. (13) suggest that Sirt3-KO mice have low bone mass. However, due to the lack of specific information about the mice (e.g., sex and age) used by Gao et al., it is unclear whether the seeming discrepancy can be explained by differences in age or genetic background. While our paper was in review, Li et al. reported that deletion of Sirt3 increases bone mass in 3- and 6-month-old female but not male mice (59). In addition, these authors show that Sirt3-KO mice are protected against OVX-induced bone loss, in line with our present findings. The different impact of Sirt3 deletion on the bone phenotype of young mice might be due to the different genetic background of the mice.

In conclusion, the results of the present work reveal that mitochondrial deacetylase Sirt3 is a major contributor of the increased bone resorption in the pathogenesis of osteoporosis. We also provide proof of principle that pharmacological inhibition of Sirt3 with LC-0296 is sufficient to mimic the skeletal effects of the genetic deletion and attenuate the loss of bone mass in aging mice. Nonetheless, these findings are at odds with previous suggestions that stimulation of Sirt3 represents a good strategy to ameliorate diseases of aging (8–11, 52). The usefulness of targeting Sirt3 in age-related diseases notwithstanding, our study highlights the importance of the regulation of mitochondria in bone cells and its contribution to diseases of the skeleton.

## Methods

### Animal experiments.

The Sirt3-KO mice were maintained and bred in our facility using pairs of mice heterozygous for the *Sirt3* mutant allele (B6/Sv129 mixed background) (The Jackson Laboratory, 012755) donated by David Gius (Northwestern University, Chicago, Illinois, USA) (24). To determine whether Sirt3 plays a role in age-associated bone loss, mice were aged up to 16 months. Six-month-old female and 4-month-old male mice of the same genotypes were used as young adult controls. To test the effects of estrogen deficiency, 5-month-old female Sirt3-KO mice and WT littermate controls were randomized into sham- or OVX-surgical groups according to their femoral DXA BMD. BMD measurements were performed 1 day prior to surgery and before euthanasia. Mice were injected with tetracycline (15 mg/kg body weight) 10 and 3 days before euthanasia to quantify bone-formation rates. Animals were sacrificed 6 weeks after surgery, and the tissues were dissected for further analyses.

To test the in vivo effects of Sirt3 antagonizing compounds, 12-month-old female C57BL/6 mice (The Jackson Laboratory) were randomly assigned to 1 of 2 treatment groups based on body weight and received i.p. injections of either the vehicle (100 μL/each injection; 1:2 DMSO/PBS mixture) or LC-0296 (5 μg/g body weight per day; AOBIOUS Inc.) 3 times per week for 16 weeks. Mice were euthanized, and tissues were harvested 2 days after the last LC-0296 administration. *ERα*^ΔLysM^** mice (C57BL/6 background) were generated as described previously (22).

Genomic DNA extracted from tail samples was used for PCR-based genotyping following the protocols from The Jackson Laboratory. All mice used in this study were housed under standard laboratory conditions with a 12-hour dark, 12-hour light cycle; a constant temperature of 23°C; and humidity of 48%. A standard rodent diet (Envigo, Teklad 22/5) containing 22% protein, 1.13% calcium, and 0.94% phosphorus was provided to mice ad libitum. For aging study, the mice were switched to Teklad 2014 rodent diet (Envigo), containing 14% protein and 4% fat, and acidified water ad libitum at 8 months of age. Investigators were blinded to study groups during animal handling and endpoint measurements.

### Soft tissue histology.

Necropsy was done in acute toxicity tests groups of animals on harvest day. After blood collection, mice were sacrificed, and the vital organs (heart, kidney, liver, and lung) were removed through a midline incision in the mouse’s abdomen. The organs were cleaned of fat, blotted with clean tissue paper, and weighed. Samples from the vital organs of both the vehicle and LC-0296 groups were subjected to histopathologic evaluation. Organ samples were fixed in 10% buffered formalin and then routinely processed and embedded in paraffin wax. Paraffin sections (5 μm) were placed on glass slides and stained with H&E. An experienced pathologist blinded to the experimental groups analyzed the sections via light microscopy (Nikon E50i, Nikon Corporation).

### DXA and micro-CT.

BMD measurements were performed by DXA using a PIXImus densitometer (GE Lunar) on mice sedated with 2% isoflurane, and data were analyzed as previously described (43). Scans of the entire left femur or lumbar spine were used for the measurement of BMD. Micro-CT analyses of the distal end of the femora and the vertebrae (L5) were performed after the bones were dissected, cleaned, fixed in Millonig’s phosphate buffer (Leica Biosystems), and gradually dehydrated in 100% ethanol. Bones were scanned with a MicroCT40 (Scanco Medical) at medium resolution (12 μm isotropic voxel size) for quantitative determinations and integrated into 3-D voxel images (1024 × 1024 pixel matrices for each planar stack). For the latter, a Gaussian filter (sigma = 0.8, support = 1) was applied to all analyzed scans. Scanco Eval Program v.6.0 was used for measuring bone volume. Scan settings included X-ray tube potential (55 kVp), X-ray intensity (145 μA), and integration time (220 ms). The nomenclature used conforms to recommendations of the American Society for Bone and Mineral Research (60). At a threshold of 200 mg/cm^3^, cortical dimensions were determined using 18–23 slices at the femoral middiaphysis and using 50 slices between slices 300 and 350 at the distal metaphysis. Total and medullary area and circumference measurements were calculated from these slices. For cortical porosity measurements, slices were analyzed from a point immediately distal to the third trochanter to the primary spongiosa. After defining endosteal and periosteal boundaries, an additional image processing script (“peel-iter =2”) was used to eliminate false voids caused by an imperfect wrap of the contours to the bone surface. Images were binarized with a threshold of 365 mg/cm^3^, and overall porosity was determined with the “cl_image” script to obtain bone volume and void volume. To avoid the inclusion of osteocyte lacunae and canalicular space, void volumes < 31,104 μm^3^ (18 voxels) were excluded in the determination of porosity. All trabecular measurements were made by drawing contours every 10–20 slices and using voxel counting for BV/TV and sphere filling distance transformation indices, without preassumptions about the bone shape as a rod or plate. Vertebral cortical bone thickness was determined on the ventral cortical wall using contours of cross-sectional images drawn to exclude trabecular bone, as described for femoral cortical bone.

### CTx, P1NP, and osteocalcin ELISA.

Circulating CTx, P1NP, and osteocalcin in serum was measured using a mouse RatLaps (CTx-I) ELISA kit (Immunodiagnostic Systems), Rat/Mouse PINP EIA kit (Immunodiagnostic Systems), and an Osteocalcin enzyme immunoassay kit (Thermo Fisher Scientific), respectively, according to the manufacturer’s directions. Blood was collected into 1.7 mL microcentrifuge tubes by retro-orbital bleeding. Blood was then kept on ice for 1 hour and centrifuged at 6,150*g* at 4°C for 10 minutes to separate serum from cells.

### Bone histology.

The terminology used in this study is that which is recommended by the Histomorphometry Nomenclature Committee of the American Society for Bone and Mineral Research (61). Freshly dissected lumbar vertebrae (L5) and femurs were fixed in 10% Millonig’s formalin overnight, followed by dehydration, embedding in methyl methacrylate, and longitudinal sectioning (5 μm). The sections were stained for TRAP activity using naphthol AS-MX and Fast Red TR salt (Sigma-Aldrich) and counterstained with toluidine blue (Sigma-Aldrich) to count osteoclasts using the OsteoMeasure Analysis System (OsteoMetrics Inc). The following dynamic measurements were made: total perimeter (B.Pm); single label perimeter (sL.Pm); double label perimeter (dL.Pm), and mineral apposition rate (MAR). The following values were then calculated: mineralizing surface (MS/BS = [1/2sL.Pm + dL.Pm]/B.Pm × 100; %); bone formation rate (BFR/BS = MAR × MS/BS; μm2/μm/day). Histomorphometry measurements of the trabecular bone were restricted to the secondary spongiosa. One section per sample was analyzed by a histopathologist blinded to the study groups.

### Cell culture.

BMMs were obtained as described previously (20, 22). Briefly, whole BM cells were flushed from the tibiae and femora. Cells from 4–5 mice of each group were pooled, depleted of RBCs with ACK buffer (0.01 mM EDTA, 0.011 M KHCO3, and 0.155 M NH4Cl [pH 7.3]), and plated in α-MEM complete media (Thermo Fisher Scientific) containing 10% FBS (Amizona Scientific), 1% penicillin and streptomycin (PSG; MilliporeSigma), and 10 ng/mL M-CSF (R&D Systems) in 10 cm culture dishes overnight. Nonadherent cells were then replated in Petri dishes with 10 ng/mL M-CSF for 4–5 days to obtain BMMs, which were used as osteoclast precursors. To generate preosteoclasts or mature osteoclasts, BMMs were cultured in α-MEM complete media with 30 ng/mL M-CSF and 30 ng/mL RANKL (R&D Systems) for 2 or 4 days. Cultured osteoclasts were fixed with 10% neutral buffered formalin for 10 minutes and stained for TRAP, using a Leukocyte Acid Phosphatase Assay Kit (Sigma-Aldrich), following the manufacturer’s instructions. A preosteoclast was defined as a round mononuclear TRAP^+^ cell, and a mature osteoclast was defined as a multinucleated (>3 nuclei) TRAP^+^ cell. Cells were plated at least in triplicate for all TRAP staining assays. To investigate the effects of estrogens on Sirt3 mRNA levels in osteoclasts, E_2_ (1 × 10^–8^M; Sigma-Aldrich) was added for 48 hours. To examine the effects of Sirt3 inhibitor on osteoclastogenesis, BMMs were isolated from 16-month-old female C57BL/6 mice and cultured with 30 ng/mL M-CSF and 30 ng/mL RANKL in the presence or absence of 10 nM LC-0296.

For osteoblast cultures, total BM cells were obtained as described above. Cells from 3–4 mice of each group were pooled and cultured with 20% FBS, 1% PSG, and 50 μg/mL ascorbic acid (Sigma-Aldrich) in 10 cm culture dishes for 7 days. Adherent BM stromal cells were trypsinized and replated in 12-well tissue culture plates at 0.15 × 10^6^ to 0.25 × 10^6^ cells per well with 10% FBS, 1% PSG, 50 μg/mL of ascorbic acid (Sigma-Aldrich), and 10 mM β-glycerophosphate (Sigma-Aldrich) for 3 days, to perform quantitative PCR (qPCR) assays and Western blotting, or for 21 days to assess mineralization. The mineralized matrix was stained with 40 mM Alizarin Red solution, following the manufacturer’s instructions (Sigma-Aldrich).

### Bone resorption assay.

BMMs were isolated as described above and stimulated with RANKL to form osteoclasts on Osteo Assay Surface 24-well plates (Corning Life Sciences) coated with an inorganic bone biomaterial surface. Cells were removed using a 2% hypochlorite solution for 5 minutes, washed with distilled water, and dried at room temperature. For Von Kossa staining, wells were treated in darkness with 150 μL/well of 5% (w/v) aqueous silver nitrate solution for 20 minutes. Plates were then washed for 5 minutes with distilled water and incubated in darkness with 150 μL/well of 5% (w/v) sodium carbonate in a 10% formalin solution. Wells were then washed twice with PBS, rinsed with distilled water, and dried in a 50°C oven for 30 minutes. Three wells per group were assessed microscopically. In this assay, the resorbed areas appear white, and the unresorbed mineralized surface appears black.

### Seahorse mitochondrial flux analysis.

The BMMs were plated and treated with 30 ng/mL RANKL for 3 days with or without 10 nM LC-0296. The media in the wells was replaced with XF assay media (Agilent), and the plate was kept in a non-CO_2_ incubator for 20 minutes at 37°C. After recording 3 total cellular respiration measurements with the XF96 analyzer (Agilent), 10 μg/mL oligomycin (Sigma-Aldrich) was added to inhibit mitochondrial ATP synthase and measure the decrease in the OCR that is linked to ATP turnover. To determine the maximal respiration potential of the cells, 10 μM FCCP (Sigma-Aldrich; an uncoupler of oxidative phosphorilation) was used. The amount of nonmitochondrial oxygen consumption was determined by inhibiting the respiratory chain activity with an antimycin A (Sigma-Aldrich) and 10 μM rotenone cocktail (Sigma-Aldrich). These data were used to calculate the mitochondrial basal respiration, ATP-linked respiration, reserve respiratory capacity, and proton leak as we previously described (22, 62).

### qPCR.

Total RNA was purified from cultured BMMs or BM stromal cells using TRIzol reagent (Thermo Fisher Scientific) according to the manufacturer’s directions. RNA was quantified using a Nanodrop instrument (Thermo Fisher Scientific), and 2 μg (cultured cells) of RNA was then used to synthesize cDNA using a High-Capacity cDNA Reverse Transcription kit (Applied Biosystems) according to the manufacturer’s instructions. Transcript abundance in the cDNA was measured by qPCR using TaqMan Universal PCR Master Mix (Thermo Fisher Scientific). The primers and probes for murine *Sirt3* (Mm00452131_m1), *Pgc1b* (Mm00504720_m1), *Tfam* (Mm00447485_m1), *Acp5* (Mm00432448_m1), *Ctsk* (Mm00484039_m1), *Itgb3* (Mm00443980_m1), *Akp* (Mm00475831_m1), *Bglap* (Mm03413826_mH), and *Runx2* (Mm00501578_m1) were manufactured by the TaqMan Gene Expression Assays service (Applied Biosystems). Relative mRNA expression levels were normalized to the house-keeping gene *ribosomal protein S2* (Mm00475528_m1) using the ΔCt method. To quantify mitochondrial DNA, total DNA was extracted from cultured cells using a QIAamp DNA Mini Kit (Qiagen), and TaqMan assays for the mitochondrial genes *ND2* (Mm04225288_s1) and *Cytb* (Mm04225271_g1) were quantified by TaqMan qPCR. Relative DNA expression levels were calculated using the transferrin receptor gene (Tfrc) copy number reference assays (Applied Biosystems) and the ΔCt method, as above.

### Quantitative analysis of mitochondrial content.

Mitochondrial content and morphology were measured using a MitoBright LT Green kit (Dojindo Molecular Technologies) according to the manufacturer’s protocol. Briefly, the BMMs were plated in 12-well tissue culture plates and treated with 30 ng/mL RANKL for 24 hours or 3 days. Cells were washed twice with α-MEM complete media, and MitoBright LT working solution was added in the cell culture plates for 30 minutes. The cells were then washed twice with Phenol red–free α-MEM and observed over time under a fluorescence microscope (Carl Zeiss Inc.). For flow cytometry analysis of mitochondria in osteoclasts, the cells were scraped, transferred to flow tubes, and analyzed over time by flow cytometry (BD LSRFortessa, BD Biosciences) with excitation at 488 nm and emission at 515 nm–545 nm.

### Western blot analysis.

Cultured cells were washed twice with ice-cold PBS and lysed with a buffer containing 20 mM Tris-HCL, 150mM NaCl, 1% Triton X-100, protease inhibitor mixture, and phosphatase inhibitor cocktail (Sigma-Aldrich) on ice for 30 minutes. The cell lysates were centrifuged at 11,200*g* for 15 minutes at 4°C, and the supernatants were collected in new tubes. The protein concentration of cell lysates was determined using a DC Protein Assay kit (Bio-Rad). The extracted protein (30–40 μg per sample) was subjected to 8%–15% SDS-PAGE gels and transferred electrophoretically onto polyvinyl difluoride membranes (MilliporeSigma). The membranes were blocked in 5% fat-free milk/Tris-buffered saline for 120 minutes and incubated with a primary antibody, followed by a secondary antibody conjugated with horseradish peroxidase. Mouse monoclonal antibodies against NFATc1 (Santa Cruz Biotechnology; sc-7294, 1:500), Mitofusin2 (Abcam; ab56889, 1:1000), Ndufa9 (Abcam; ab14713, 1:1000), Bnip3 (Abcam; ab10433, 1:1000), and Pink1 (Santa Cruz Biotechnology; sc-517353, 1:500) were used to detect their corresponding protein. We also used rabbit polyclonal antibodies for c-Fos (Santa Cruz Biotechnology; sc-7202, 1:500), LC3 (Sigma-Aldrich; L8918, 1:1000), and p62 (Sigma-Aldrich; P0067, 1:1000). Rabbit monoclonal antibodies against Sirt3 (Cell Signaling Technology; 5490, 1:1000), HSP60 (Cell Signaling; 12165, 1:1000), SDHA (Cell Signaling Technology; 11998, 1:1000), VDAC (Cell Signaling Technology; 4661, 1:1000), CoxIV (Cell Signaling Technology; 4850, 1:1000), pyruvate dehydrogenase (Cell Signaling Technology; 3205, 1:1000), and Nix (Cell Signaling Technology; 12396, 1:1000) were used to detect Sirt3 and the other representative mitochondria proteins. Polyclonal antibodies against Ac-Pink1 were produced in rabbits in cooperation with Creative Biolabs Inc (1:1000). Ac-Pink1 antibodies bind to 1 of the 2 acetylated lysines or both. Blots were stripped and reprobed with anti–β-actin antibody (Santa Cruz Biotechnology; sc-81178, 1:2000). Bound antibodies were detected with ECL reagents (MilliporeSigma) and were imaged and quantified with a VersaDoc imaging system (Bio-Rad). See complete unedited blots in the supplemental material.

### Statistics.

All data were analyzed using GraphPad Prism 8 or 9 (GraphPad Software). Statistically significant treatment effects were detected with a 2-way ANOVA after determining that the data were normally distributed and exhibited equivalent variances. In some cases, log or rank transformations were used to obtain normally distributed data and equal variance. This was followed by pairwise comparisons using Tukey’s procedure. For experiments involving a comparison of only 2 groups, a 2-tailed Student’s *t* test was used. *P* < 0.05 was considered significant.

### Study approval.

The IACUC of the University of Arkansas for Medical Sciences and the Central Arkansas Veterans Healthcare System reviewed and approved all studies involving mice.

## Author contributions

HNK, MA, and NAB conceived and designed the experiments. WL, KK, and ADW generated mice. HNK, WL, KK, KKR, ADW, and FP performed the experiments. HNK, WL, FP, NAB, SCM, and MA analyzed the results. HNK and MA prepared the manuscript with the assistance of SCM. All authors reviewed the manuscript.

## Supplementary Material

Supplemental data

## Version 1 04/20/2021

In-Press Preview

## Version 2 05/24/2021

Electronic publication
