# Supplementary material for: Mitochondrial Sirt3 contributes to the bone loss caused by aging or estrogen deficiency
Source: JCI Insight. 2021 May 24;6(10):e146728. doi: 10.1172/jci.insight.146728 (PMC8262324; doi:10.1172/jci.insight.146728)
Supplement: Supplemental data [file jciinsight-6-146728-s123.pdf]

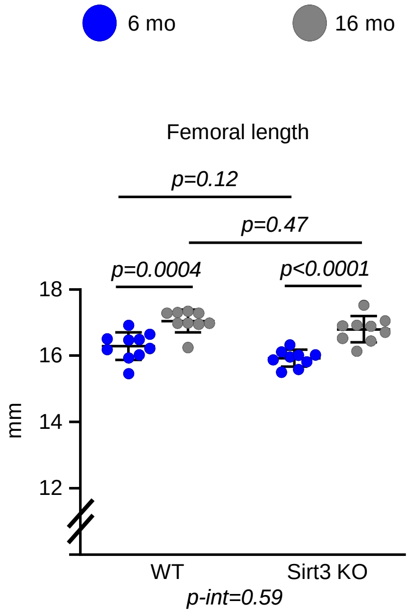

Supplementary Figure 1. Deletion of Sirt3 does not affect femoral length.
The graph indicates their femoral lengths in young and aged group. Lines and error bars represent mean ± SD. *P* values were determined using 2-way ANOVA. Interaction terms generated by 2-way ANOVA analysis are shown below each graph.

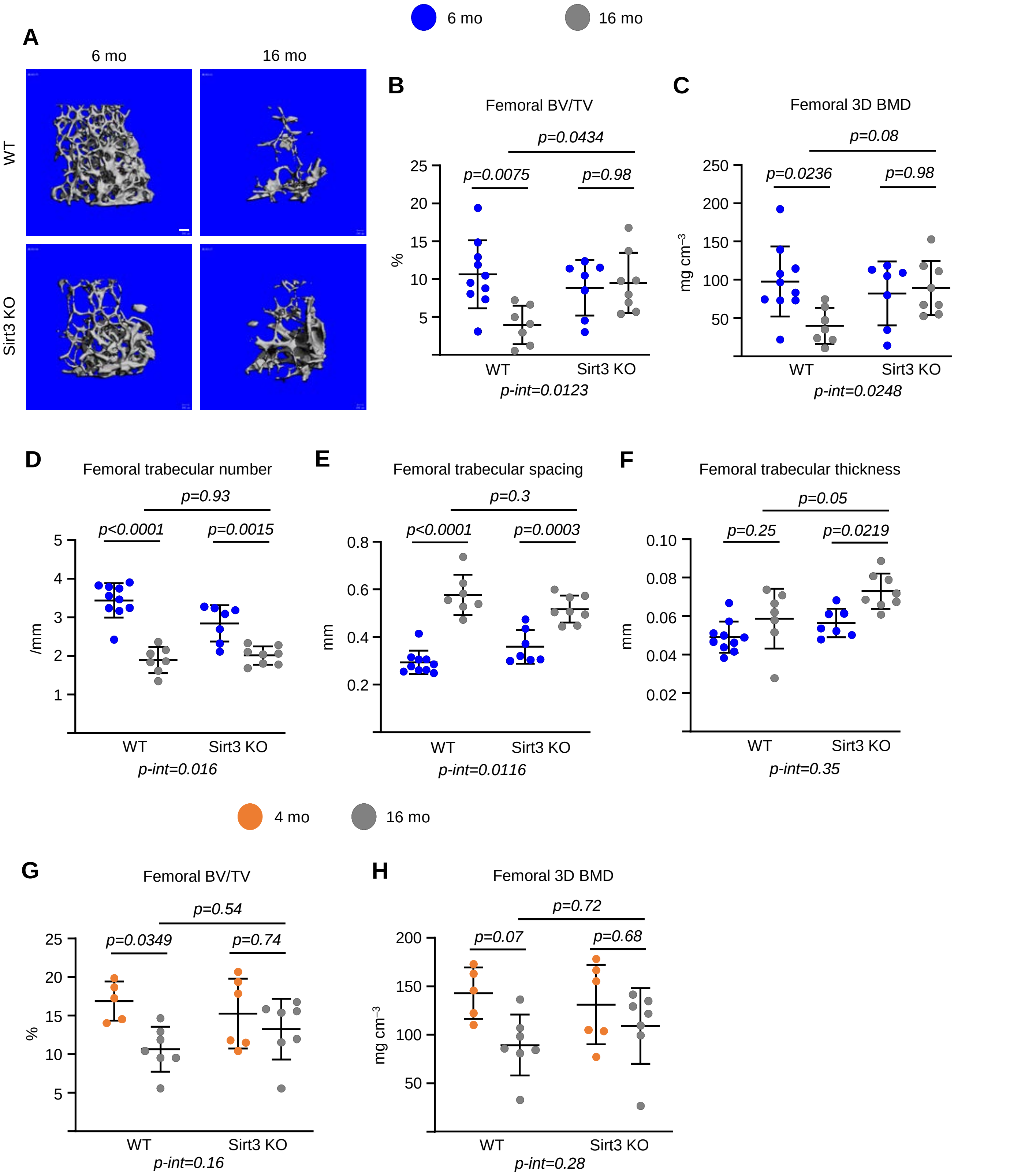

Supplementary Figure 2. Deletion of Sirt3 prevents age-associated trabecular bone loss at the femur.
(**A**–**F**) Imaging and quantification of femoral bones from female Sirt3 knockout mice and wild-type littermates by micro-CT after sacrifice (*n* = 7–10 animals/group). (**A**) Representative images of trabecular bone (scale bar: 100 µm) and (**B**–**F**) bone volume per tissue volume (BV/TV), bone mineral density (BMD), and microarchitecture of trabecular bone in femur. (**G**–**H**) BV/TV and BMD of trabecular bone in femur from male Sirt3 knockout mice and wild-type littermates by micro-CT (*n* = 5–7 animals/group). Lines and error bars represent mean ± SD. *P* values were determined using 2-way ANOVA. Interaction terms generated by 2-way ANOVA analysis are shown below each graph.

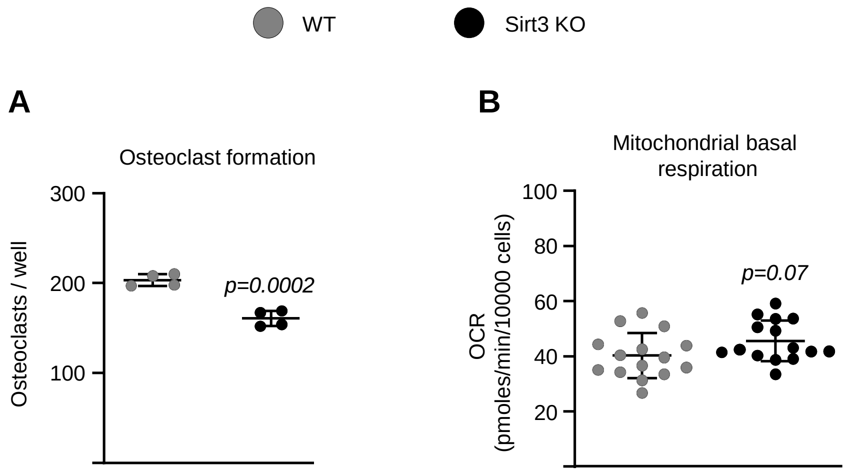

Supplementary Figure 3. Deletion of Sirt3 does not affect mitochondrial respiration of osteoclasts in young mice.
BMMs were isolated from 6-month-old female Sirt3 knockout mice and wild-type littermate controls and cultured with M-CSF (30 ng/mL) and RANKL (30 ng/mL) for (**A**) 5 or (**B**) 3 days. (**A**) Number of TRAP-positive multinucleated osteoclasts generated from BMMs (quadruplicates of pooled cultures). (**B**) Mitochondrial respirations per cell, in osteoclasts, measured by Seahorse (*n* = 15 wells/group). Lines and error bars represent mean ± SD. *P* values were determined using Student's *t*-test. All measures were performed in cultured BMMs pooled from 4-5 mice/group.

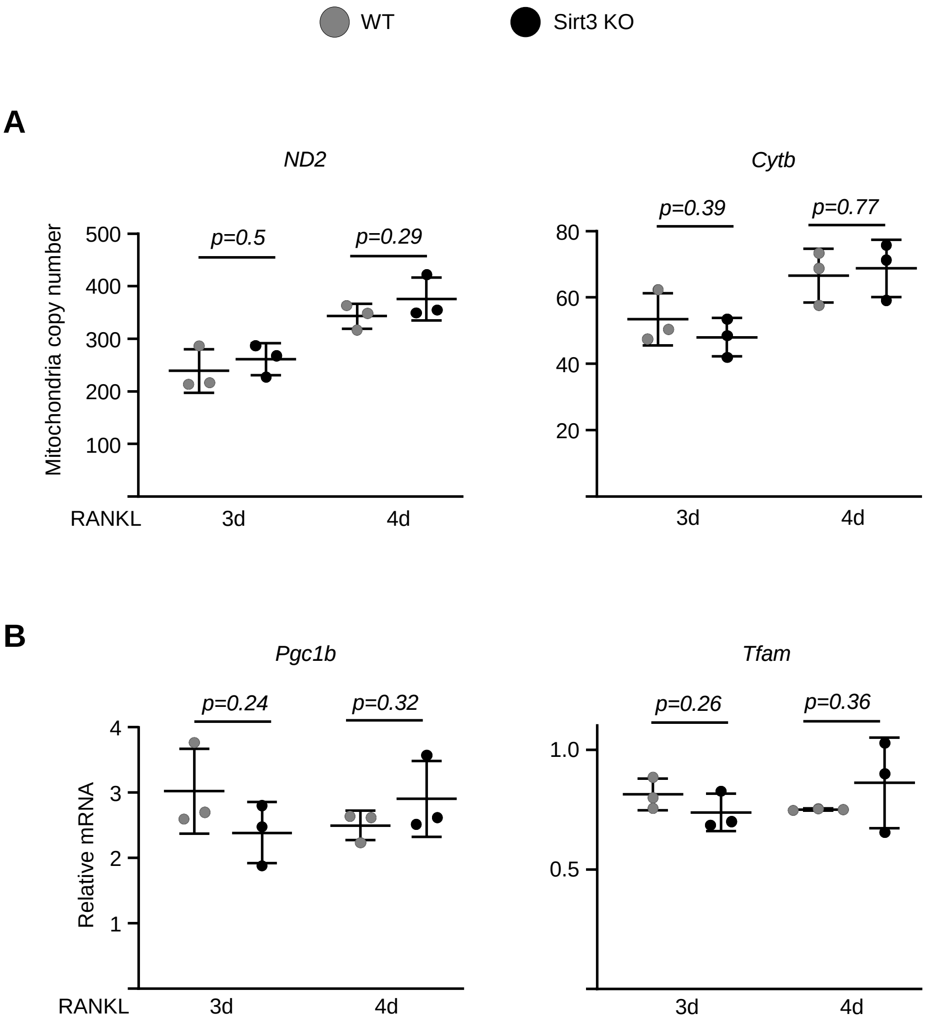

Supplementary Figure 4. Deletion of Sirt3 does not affect mitochondrial biogenesis of osteoclasts in aged mice.
BMMs were isolated from 16-month-old female Sirt3 knockout mice and wild-type littermate controls and cultured with M-CSF (30 ng/mL) and RANKL (30 ng/mL) for indicated times. (**A**) Ratio of mitochondrial to nuclear DNA and (**B**) mRNA levels of *Pgc1b* and *Tfam* in osteoclasts measured by qRT-PCR. *P* values were determined using Student's *t*-test. All measures were performed in cultured BMMs pooled from 4-5 mice/group.

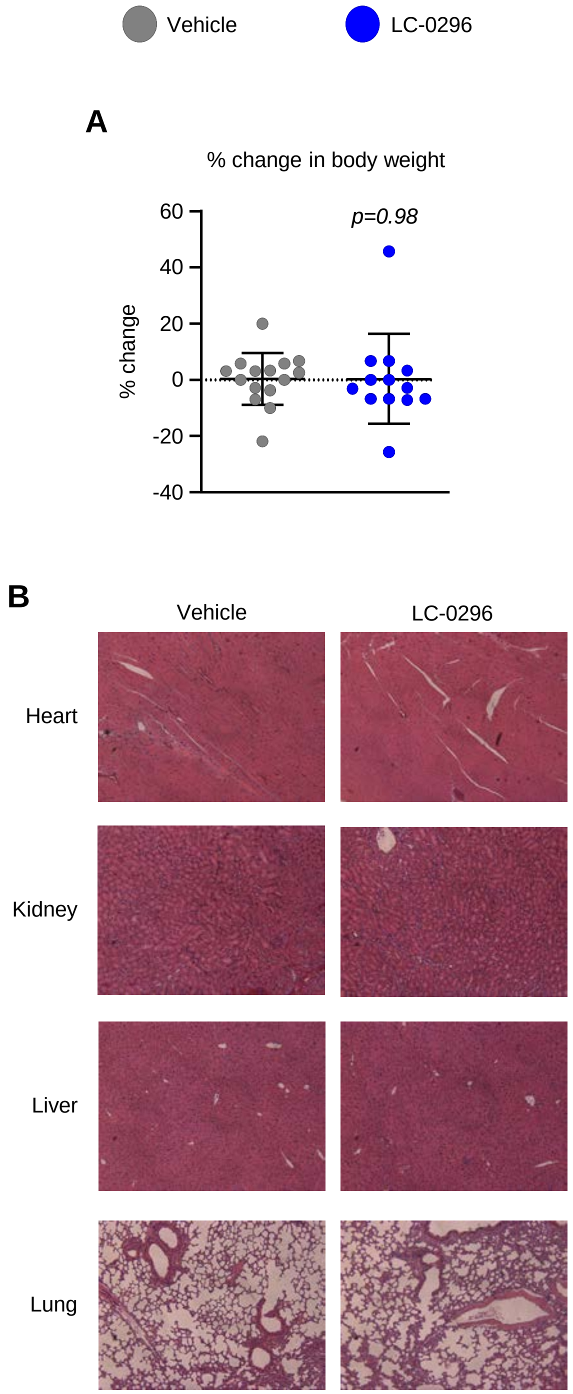

Supplementary Figure 5. Administration of LC-0296 was not toxic to aging mice.
LC-0296 was administrated (5 µg/g body weight, 100 uL/each i.p. injection) into 12-month-old female C57BL/6 mice for 4 months. (**A**) Percent change in body weight determined 1 day before LC-0296 injection and before sacrifice. (**B**) Representative sections of vital organs from aging mice untreated and treated with LC-0296 following H&E staining as observed under a light microscope (200x). *P* values were determined using Student's *t*-test.

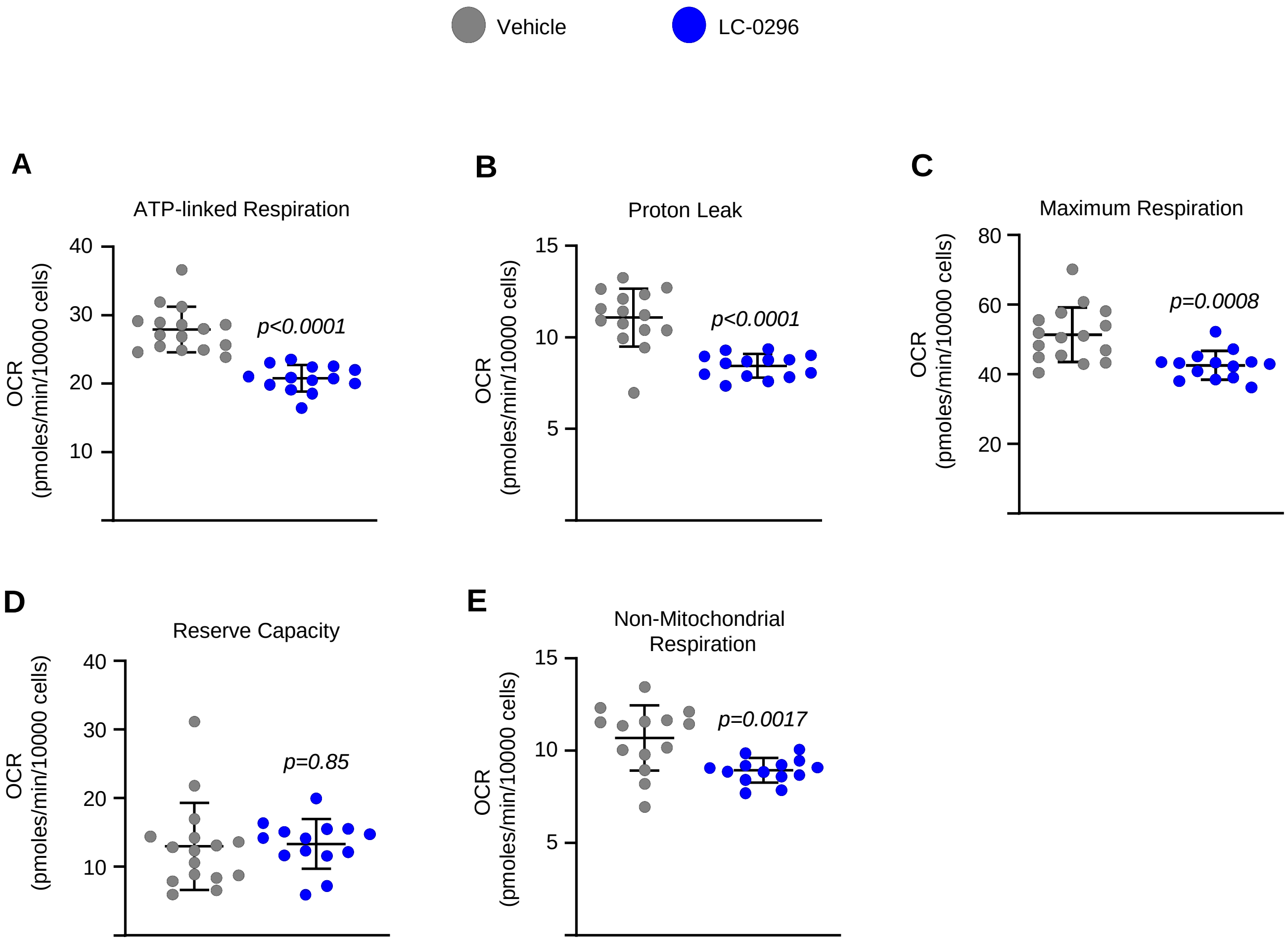

Supplementary Figure 6. Inhibition of Sirt3 by LC-0296 attenuates mitochondrial and non-mitochondrial respiration in osteoclasts of aging mice.
(**A**–**E**) BMMs were isolated from 16-month-old female C57BL/6 mice and cultured with M-CSF (30 ng/mL) and RANKL (30 ng/mL) for 3 days in the presence or absence of LC-0296 (10 nM). Different fractions of mitochondrial and non-mitochondrial respirations per cell, in osteoclasts, measured by Seahorse (*n* = 14–16 wells/group). Lines and error bars represent mean ± SD. *P* values were determined using Student's *t*-test.

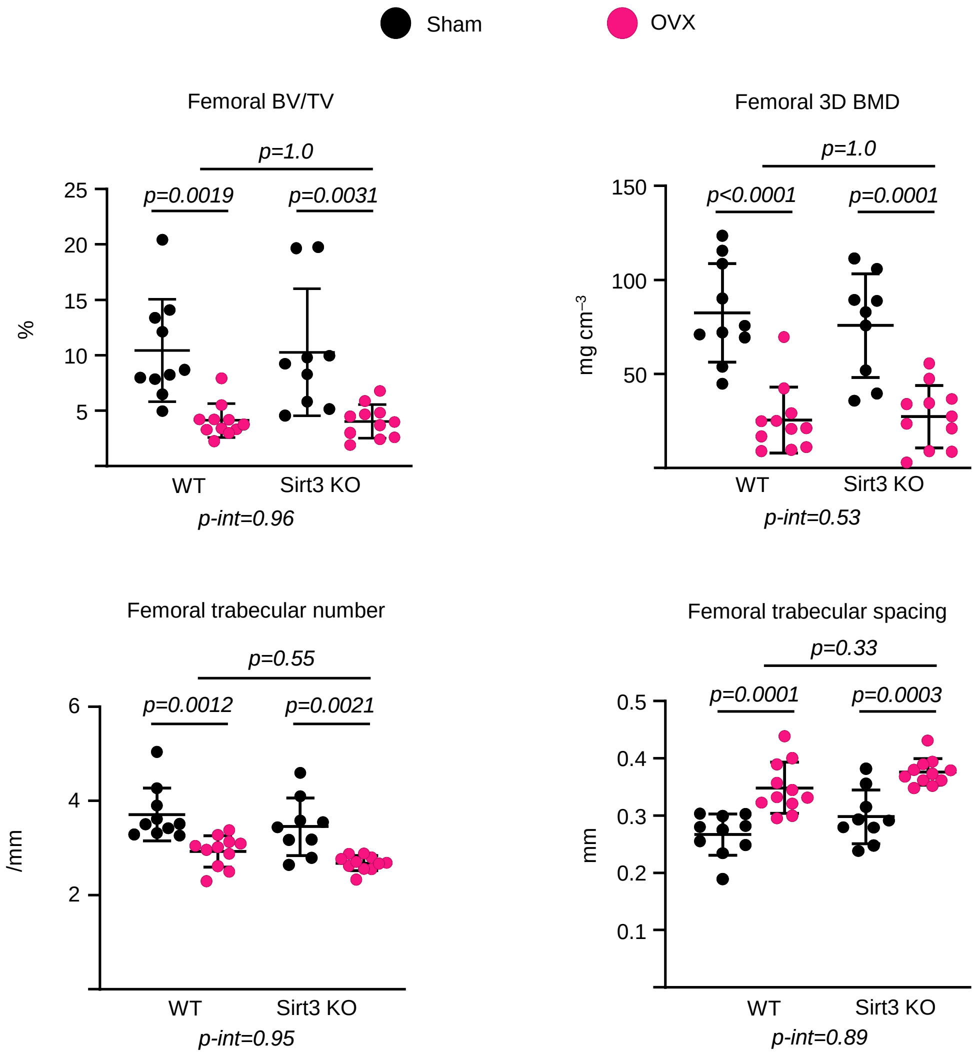

Supplementary Figure 7. Deletion of Sirt3 does not affect trabecular bone of femur in OVXed mice.
Five-month-old female Sirt3 knockout mice and wild-type littermate controls were sham-operated or ovariectomized (OVX) for 6 weeks. Bone volume per tissue volume (BV/TV), bone mineral density (BMD), and microarchitecture of trabecular bone in femur by micro-CT (*n* = 9–11 animals/group). Lines and error bars represent mean ± SD. *P* values were determined using 2-way ANOVA. Interaction terms generated by 2-way ANOVA analysis are shown below each graph.

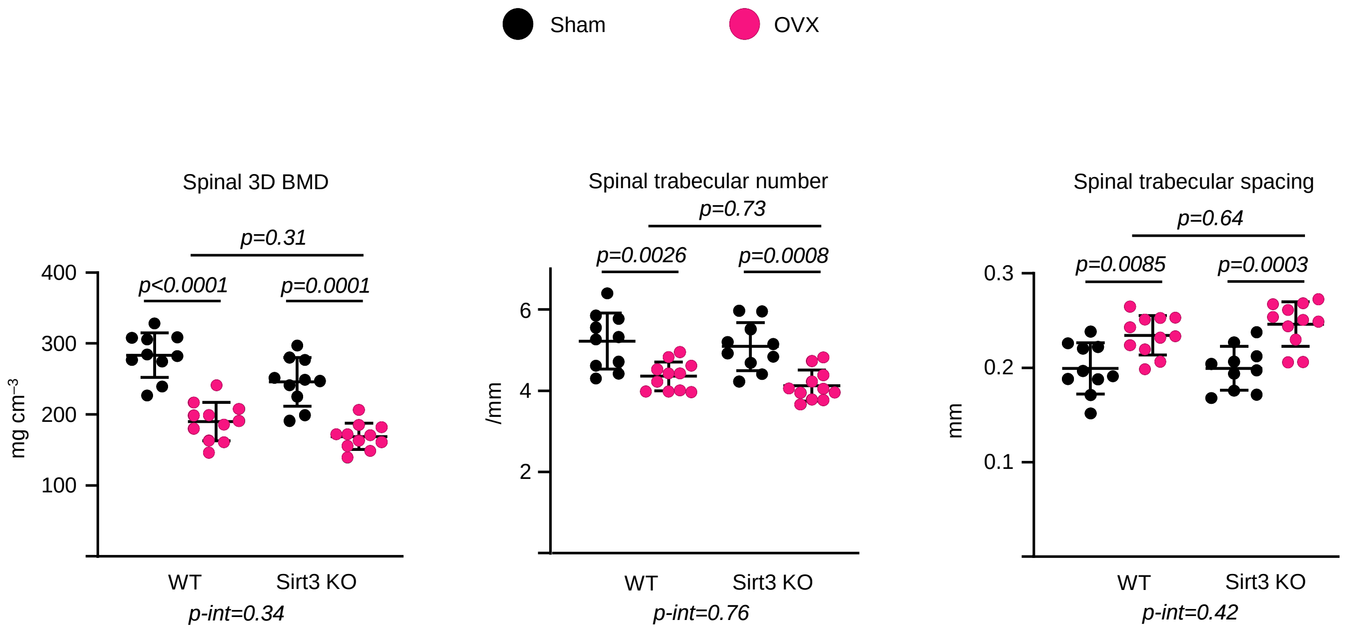

Supplementary Figure 8. Deletion of Sirt3 does not affect trabecular bone of vertebrae in OVXed mice.
Bone mineral density (BMD) and microarchitecture of trabecular bone in vertebrae after OVX by micro-CT (*n* = 9–11 animals/group). Lines and error bars represent mean ± SD. *P* values were determined using 2-way ANOVA. Interaction terms generated by 2-way ANOVA analysis are shown below each graph.

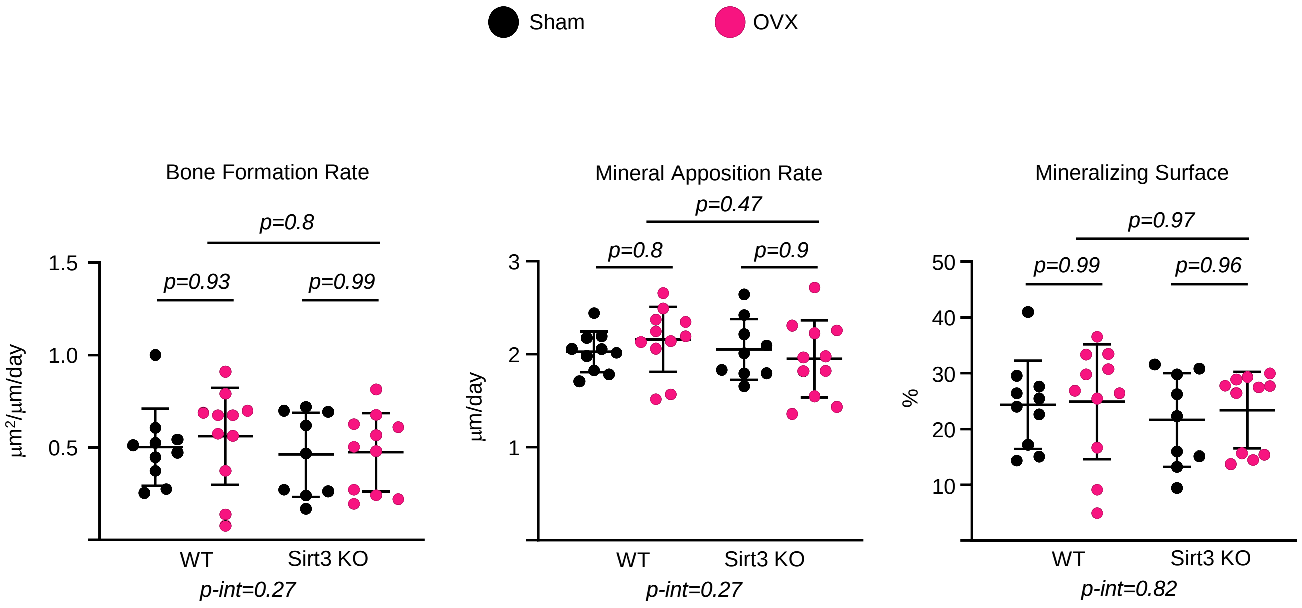

Supplementary Figure 9. Deletion of Sirt3 does not affect bone formation of femur in OVXed mice.
Bone formation rate, Mineral apposition rate and mineralizing surface in the endocortical surface of Sirt3 knockout mice and wild-type littermates after OVX (n=9–11 animals/group). Lines and error bars represent mean ± SD. *P* values were determined using 2-way ANOVA. Interaction terms generated by 2-way ANOVA analysis are shown below each graph.
